# Design, synthesis, in vitro, and in silico anti-α-glucosidase assays of *N*-phenylacetamide-1,2,3-triazole-indole-2-carboxamide derivatives as new anti-diabetic agents

**DOI:** 10.1038/s41598-024-66201-y

**Published:** 2024-07-09

**Authors:** Mohammad Hossein Sayahi, Samira Zareei, Mohammad Halimi, Majid Alikhani, Ali Moazzam, Maryam Mohammadi-Khanaposhtani, Somayeh Mojtabavi, Mohammad Ali Faramarzi, Hossein Rastegar, Parham Taslimi, Essam H. Ibrahim, Hamed A. Ghramh, Bagher Larijani, Mohammad Mahdavi

**Affiliations:** 1 Department of Chemistry, Payame Noor University, Tehran, Iran; 2 Endocrinology and Metabolism Research Center, Endocrinology and Metabolism Clinical Sciences Institute, Tehran University of Medical Sciences, Tehran, Iran; 3 Department of Biology, Babol Branch, Islamic Azad University, Babol, Iran; 4 Department of Internal Medicine, School of Medicine, Rheumatology Research Center Shariati Hospital, Tehran University of Medical Sciences, Tehran, Iran; 5 Cellular and Molecular Biology Research Center, Health Research Institute, Babol University of Medical Sciences, Babol, Iran; 6 Department of Pharmaceutical Biotechnology, Faculty of Pharmacy, Tehran University of Medical Sciences, Tehran, Iran; 7 Cosmetic Products Research Center, Iranian Food and Drug Administration, MOHE, Tehran, Iran; 8 Department of Biotechnology, Faculty of Science, Bartin University, Bartin, Turkey; 9 Biology Department, Faculty of Science, King Khalid University, P.O. Box 9004, 61413 Abha, Saudi Arabia; 10 Research Center for Advanced Materials Science (RCAMS), King Khalid University, Abha, Saudi Arabia; 11 Blood Products Quality Control and Research Department, National Organization for Research and Control of Biologicals, Cairo, Egypt; 12 Unit of Bee Research and Honey Production, Faculty of Science, King Khalid University, 61413 Abha, Saudi Arabia

**Keywords:** Indole, Carboxamide, 1,2,3-Triazole, *N*-Phenylacetamide, α-Glucosidase inhibitor, Computational biology and bioinformatics, Drug discovery, Structural biology, Molecular medicine

## Abstract

In this work, a novel series of *N*-phenylacetamide-1,2,3-triazole-indole-2-carboxamide derivatives **5a–n** were designed by consideration of the potent α-glucosidase inhibitors containing indole and carboxamide-1,2,3-triazole-*N*-phenylacetamide moieties. These compounds were synthesized by click reaction and evaluated against yeast α-glucosidase. All the newly title compounds demonstrated superior potency when compared with acarbose as a standard inhibitor. Particularly, compound **5k** possessed the best inhibitory activity against α-glucosidase with around a 28-fold improvement in the inhibition effect in comparison standard inhibitor. This compound showed a competitive type of inhibition in the kinetics. The molecular docking and dynamics demonstrated that compound **5k** with a favorable binding energy well occupied the active site of α-glucosidase.

## Introduction

α-Glucosidase is a digestive enzyme that belongs to a category of glycoside hydrolases. This enzyme liberates glucose of oligosaccharides and disaccharides by cleaving the glycosidic bond^1–3^. The most important role of this enzyme in the body is absorption of dietary carbohydrates by breaking them to glucose. Therefore, preventing the breakdown of carbohydrates by inhibition of α-glucosidase is a treatment method for type 2 diabetes^4–6^. On the other hand, α-glucosidase play an important role in maturation of glycoprotein and its folding^7^. This action of α-glucosidase has a special importance in treatment of other diseases such viral infection and cancer^8–10^. There are drugs with α-glucosidase inhibition mechanism for treatment of type 2 diabetes such as acarbose, emiglitate, miglitol, voglibose, but due to their low potency and digestive side effects, development of α-glucosidase inhibitor is still an important goal for medicinal chemists^11^.

**Figure 1:**
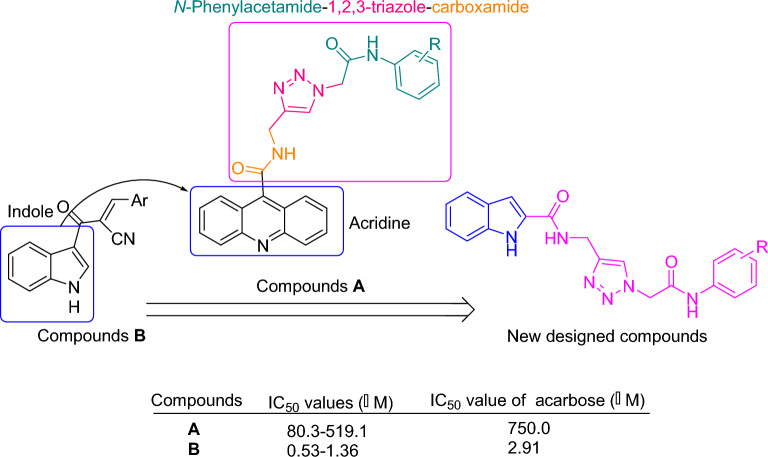
Design strategy for *N*-phenylacetamide-1,2,3-triazole-indole-2-carboxamide scaffold as the new α-glucosidase inhibitor.

Recently, many series of α-glucosidase inhibitors containing 1,2,3-triazole ring have been reported^12^. Based on the construction of the 1,2,3-triazole ring by the click reaction is a valuable tool in connecting effective pharmacophores to each other, by using this property of click reaction, effective compounds against α-glucosidase were obtained^12^. Moreover, the modified structures based on 1,2,3-triazole ring such as benzyl-1,2,3-triazole, phenoxy-1,2,3-triazole, and 1,2,3-triazole*-N*-phenylacetamide moieties were also observed in the potent α-glucosidase inhibitors^13,14^. In addition to the latter mentioned modified structures, *N*-phenylacetamide-1,2,3-triazole-carboxamide moiety also was fund in potent α-glucosidase inhibitors **A** (Fig. 1)^15^. These α-glucosidase inhibitors have an acridine ring in their structures. Acridine ring and other nitrogen containing heterocycles are very popular in the design of biologically active compounds^16,17^. One of the most popular of these rings, especially in the design of α-glucosidase inhibitors, is indole ring^18^. Various types of synthetic indole-based α-glucosidase inhibitors such as compounds **B** had been introduced^19^.

As can be seen in Fig. 1, the successful synthesis of acridine-9-carboxamide-1,2,3-triazole-*N*-phenylacetamide derivatives and the effectiveness of these compounds against α-glucosidase encouraged us to use indole as a popular α-glucosidase inhibitor pharmacophore instead of acridine^18^. As a result, *N*-phenylacetamide-1,2,3-triazole-indole-2-carboxamide scaffold was designed and fourteen derivatives of it were synthesized (Fig. 1). These derivatives carefully evaluated in vitro and in silico.

## Results and discussion

### Chemistry

**Scheme 1:**
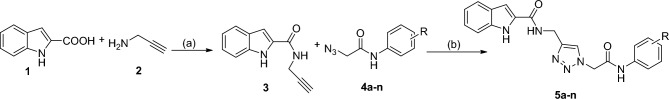
Synthesis of *N*-phenylacetamide-1,2,3-triazole-indole-2-carboxamide derivatives **5a–n**: (**a**) TBTU, Et_3_N, DMF, RT, 24 h; (**b**) CuSO_4_.5H_2_O, sodium ascorbate, RT, 18–24 h.

In this work, we synthesized a novel series of *N*-phenylacetamide-1,2,3-triazole-indole-2-carboxamide derivatives **5a–n** by click reaction. As outlined in Scheme 1, *N*-(prop-2-yn-1-yl)-1*H*-indole-2-carboxamide **3**, as a participant component in click reaction was produced by reaction between 1*H*-indole-2-carboxylic acid **1** and propargylamine **2** in the presence of TBTU and Et_3_N. Compound **3** and azide derivatives **4a–n** converted to target compounds **5a–n** by a click reaction^15^.

**Scheme 2:**
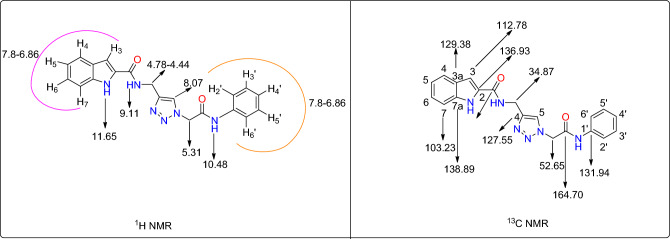
NMR interpretation of compound **5a** (unit of numbers is ppm).

For example, ^1^H NMR and ^13^C NMR interpretations of the selected compound **5a** were depicted in Scheme 2.

### Anti-α-glucosidase assay and SAR survey

Inhibitory activities of the new *N*-phenylacetamide-1,2,3-triazole-indole-2-carboxamide derivatives **5a–n** were evaluated against yeast form of α-glucosidase and the observed data were compared to acarbose as a standard inhibitor of this enzyme. As evidenced by structures and inhibition data of title compounds **5a–n** that listed in Table 1, all derivatives of *N*-phenylacetamide-1,2,3-triazole-indole-2-carboxamide scaffold with IC_50_ values ranging from 26.8 ± 0.5 to 311.3 ± 2.4 μM are excellent α-glucosidase inhibitor when compared to standard inhibitor with IC_50_ value of 752.0 ± 2.0 μM.

**Table 1:** Structures and IC_50_ values of *N*-phenylacetamide-1,2,3-triazole-indole-2-carboxamide derivatives **5a–n** against α-glucosidase.


Compound	R	IC_50_ (µM)
**5a**	H	62.9 ± 0.3
**5b**	3-Me	63.6 ± 1.1
**5c**	4-Me	42.6 ± 0.9
**5d**	2,3-Dimethyl	66.0 ± 1.8
**5e**	2,6-Dimethyl	110.8 ± 1.6
**5f**	4-Et	40.2 ± 1.6
**5g**	4-OMe	83.0 ± 0.7
**5h**	3-Cl	311.3 ± 2.4
**5i**	4-Cl	83.0 ± 1.6
**5j**	2,4-Dichloro	39.6 ± 1.4
**5k**	4-Br	26.8 ± 0.5
**5l**	3-NO_2_	110.3 ± 0.6
**5m**	4-NO_2_	78.8 ± 0.3
**5n**	2-Me-4-NO_2_	43.5 ± 0.7
Acarbose	–	752.0 ± 2.0

Based on the structure–activity relationship (SAR) screen, the strongest and weakest derivatives were halo-substituted compounds: 4-bromo derivative **5k** as the most potent compound and 3-chloro derivative **5h** as the less potent compound. Replacement of 3-chloro substituent of compound **5h** with 3-nitro group as in the case of compound **5l** created a significant increase in inhibition effect. Moreover, changing the position of nitro group from 3-position of compound **5l** to 4-position as in the case of compound **5m**, led to a moderate increase in the inhibition effect. Moreover, introduction of a methyl group on 2-position of phenyl ring of *N*-phenylacetamide moiety of 4-nitro derivative **5m** as in the case of 2-methyl-4-nitro derivative **5n** increased inhibitory activity against α-glucosidase.

The second potent compound was 2,4-dichloro derivative **5j**. The inhibitory activity of this compound reduced dramatically when the 2-chloro was removed from phenyl ring of *N*-phenylacetamid moiety as in the case of 4-chloro derivative **5i**. On the other hand, the replacement of the 4-chloro substituent of compound **5i** with methoxy group did not change inhibition effect. In contrast, the replacement of 4-chloro substituent of compound **5i** with ethyl or methyl group led to a significant increase in the inhibitory activity as observed in 4-ethyl derivative **5f** and 4-methyl derivative **5c**.

SAR study also demonstrated that un-substituted compound **5a** and 3-methyl derivative **5b** showed almost the same effects. Introduction of the second methyl group in 2-position of 3-methyl derivative **5b** as in the case of 2,3-dimethyl derivative **5d** led to a negligible decrease in inhibition effect. On the other hand, replacement of 3-methyl group in compound **5d** to 6-position as in the case of 2,6-dimethyl derivative **5e** created a significant decrease in anti-α-glucosidase activity.

**Figure 2:**
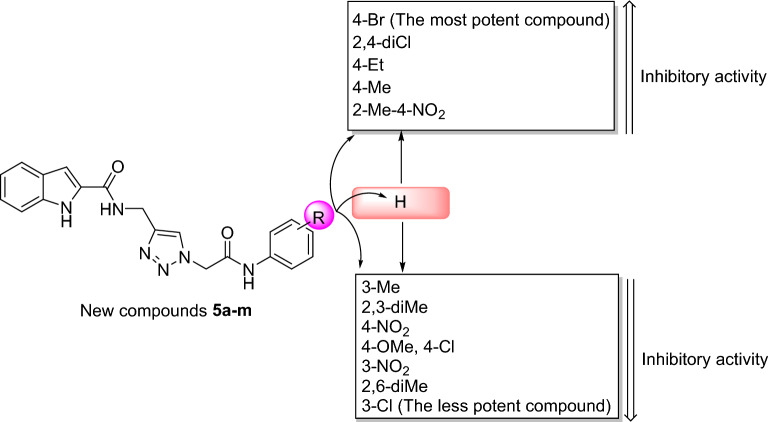
The comparison of anti-α-glucosidase activity of un-substituted compound **5a** with substituted compounds **5b–n**.

Moreover, the comparison of inhibitory activity of the substituted compounds **5b–n** with un-substituted compound **5a** depicted in Fig. 2. This figure showed that introduction of 4-Br, 2,4-dichloro, 4-ethyl, 4-methyl, and 2-methyl-4-nitro substituents on the phenyl ring of *N*-arylacetamide moiety improved inhibition effect in comparison to un-substituted compound **5a** while the presence of 3-methyl, 2,3-dimethyl, 4-nitro, 4-methoxy, 4-chloro, 3-nitro, 2,6-dimethyl, and 3-chloro substituents deteriorated anti-α-glucosidase activity.

**Figure 3:**
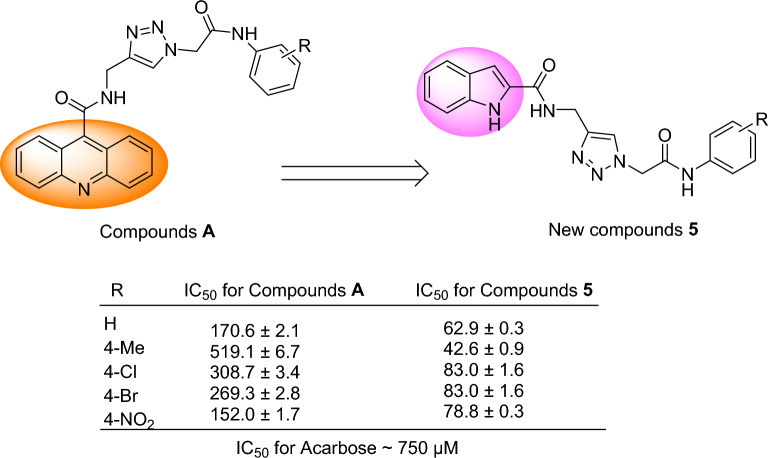
α-Glucosidase inhibitory activity of the template compounds **A** in comparison to their corresponding analogs of new compounds **5**.

The comparison of IC_50_ values of the new *N*-phenylacetamide-1,2,3-triazole-indole-2-carboxamide derivatives **5** with their corresponding analogs of acridin-9-carboxamide-1,2,3-triazole-*N*-phenylacetamide derivatives **A** is showed in Fig. 3^15^. As can be seen in this figure, all derivatives of the new compounds** 5** were more potent than their analogs of **A** series.

### Kinetics

**Figure 4:**
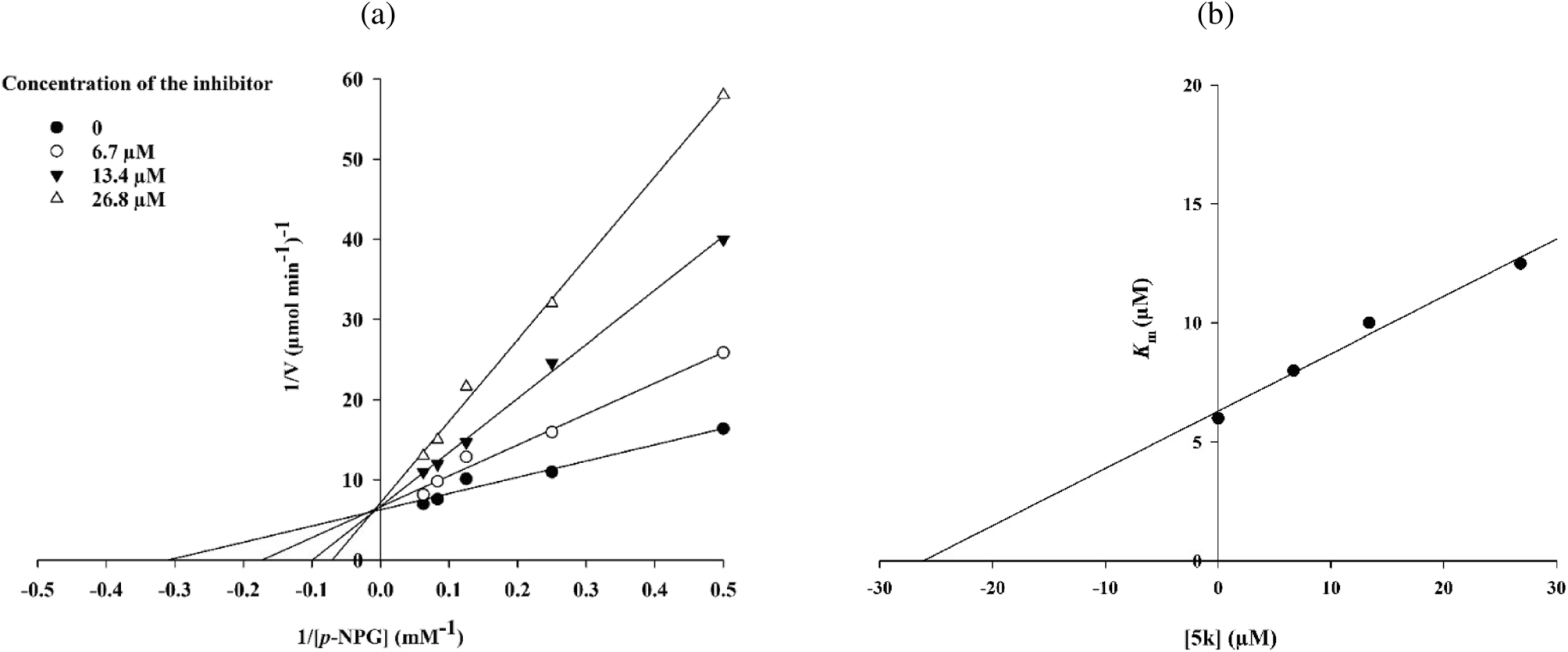
Kinetics of the new potent α-glucosidase inhibitor **5k**.

Kinetics of α-glucosidase inhibition performed on the compound **5k** as the most potent compound. As shown in Fig. 4a, the lines of Lineweaver–Burk plot with enhancement the concentration of compound **5k** had a fixed intercept on the Y-intercept and X-slopes. As the result, with increasing concentration of compound **5k**, V_max_ values remained constant while K_m_ values increased. The latter obtained result demonstrated that the most potent new compound was a competitive inhibitor (Fig. 4a). Furthermore, according to Fig. 4b (secondary plot of Lineweaver–Burk plots), the K_i_ value of compound **5k** was 26.0 µM.

### α-Amylase assay

Inhibitory activity of the most poten new α-glucosidase inhibitors **5k**, **5j**, and **5f** was evaluated against α-amylase (pancreatic form)^15^. In vitro inhibition assay of compounds **5k**, **5j**, and **5f** demonstrated that these compounds with IC_50_ value > 150 μM were inactive against α-amylase when compared with positive control acarbose with IC_50_ of 108 ± 0.71 μM. Considering that most of the side effects associated with marketed α-glucosidase inhibitors are due to their inhibitory effect on α-amylase, this feature can be considered an advantage for our new compounds^11^.

### Molecular docking study

**Figure 5:**
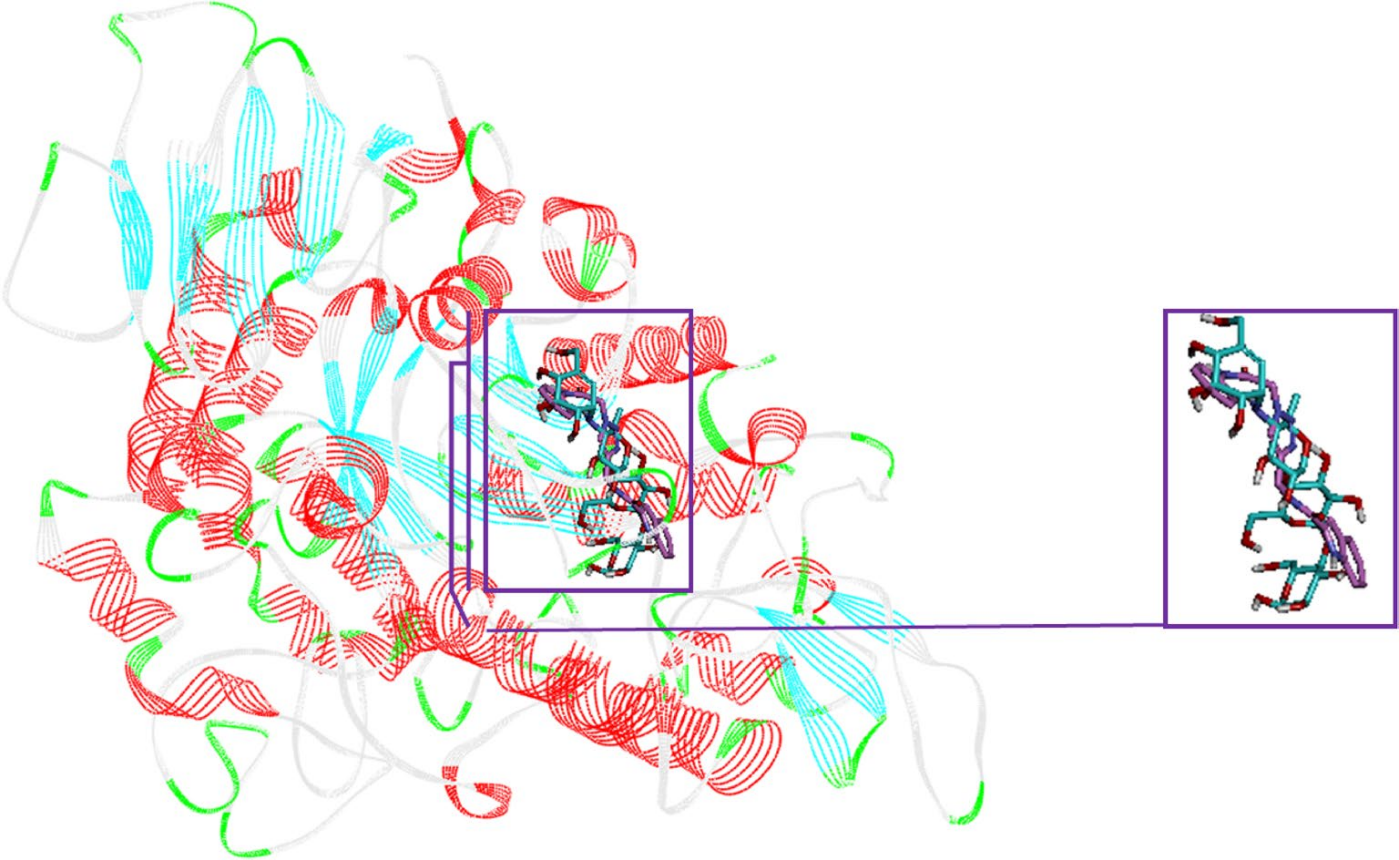
Acarbose (cyan) and the most potent compound **5k** (pink) superimposed in the α-glucosidase active site.

In order to explain interaction modes of the newly synthesized compounds in the α-glucosidase active site and rationalize observed SAR, molecular docking study carried out. Reliability of docking process was validated by re-docking of acarbose over modeled α-glucosidase and obtained RMSD value was 1.7 Å that is in the acceptable range (< 0.2). The superposed structure of standard inhibitor (acarbose) and the most potent new compound (compound **5k**) in the active site of α-glucosidase was shown in Fig. 5.

**Figure 6:**
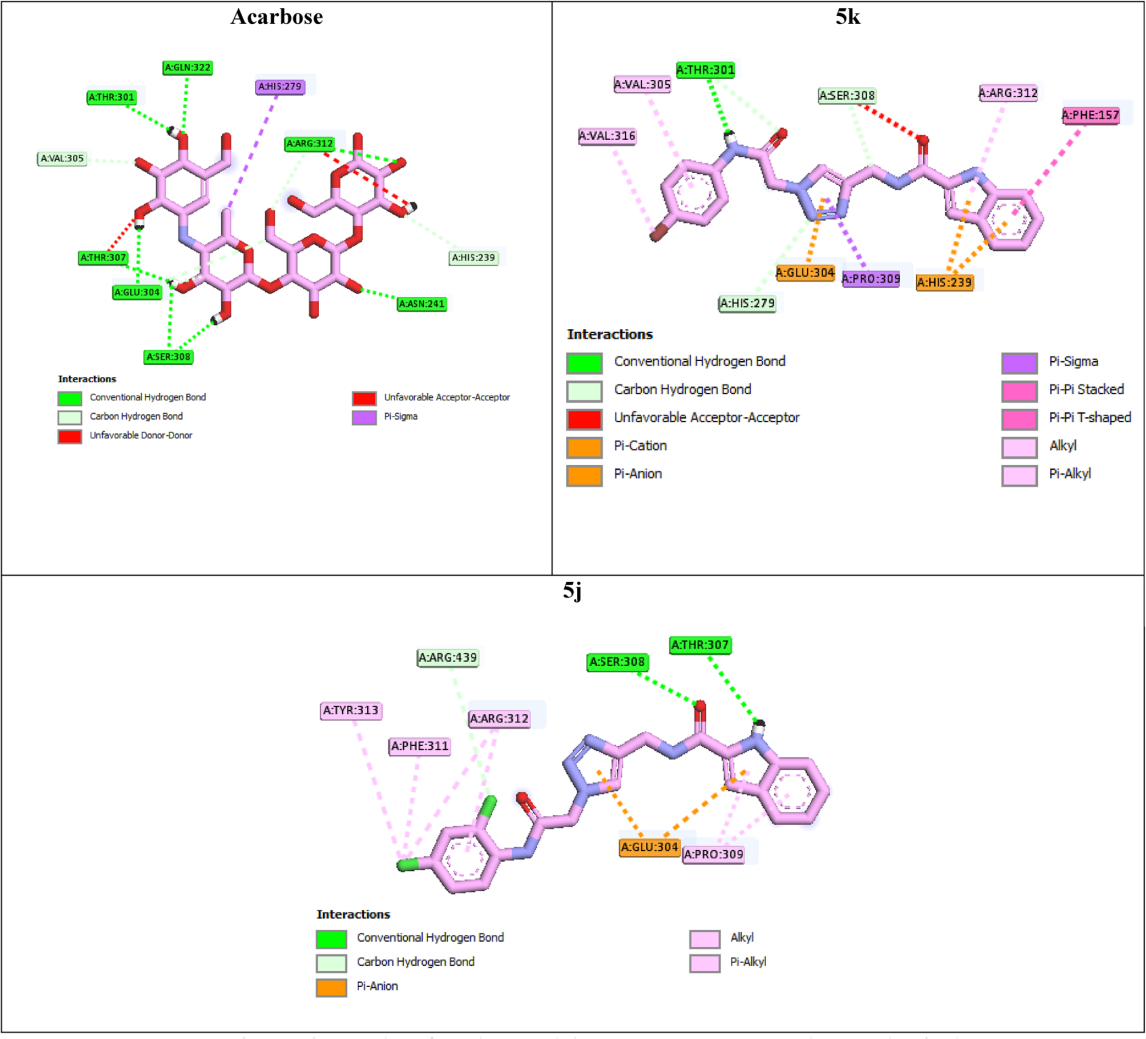
2D interaction modes of acarbose and the most potent compounds **5k** and **5j** in the α-glucosidase active site.

**Figure 7:**
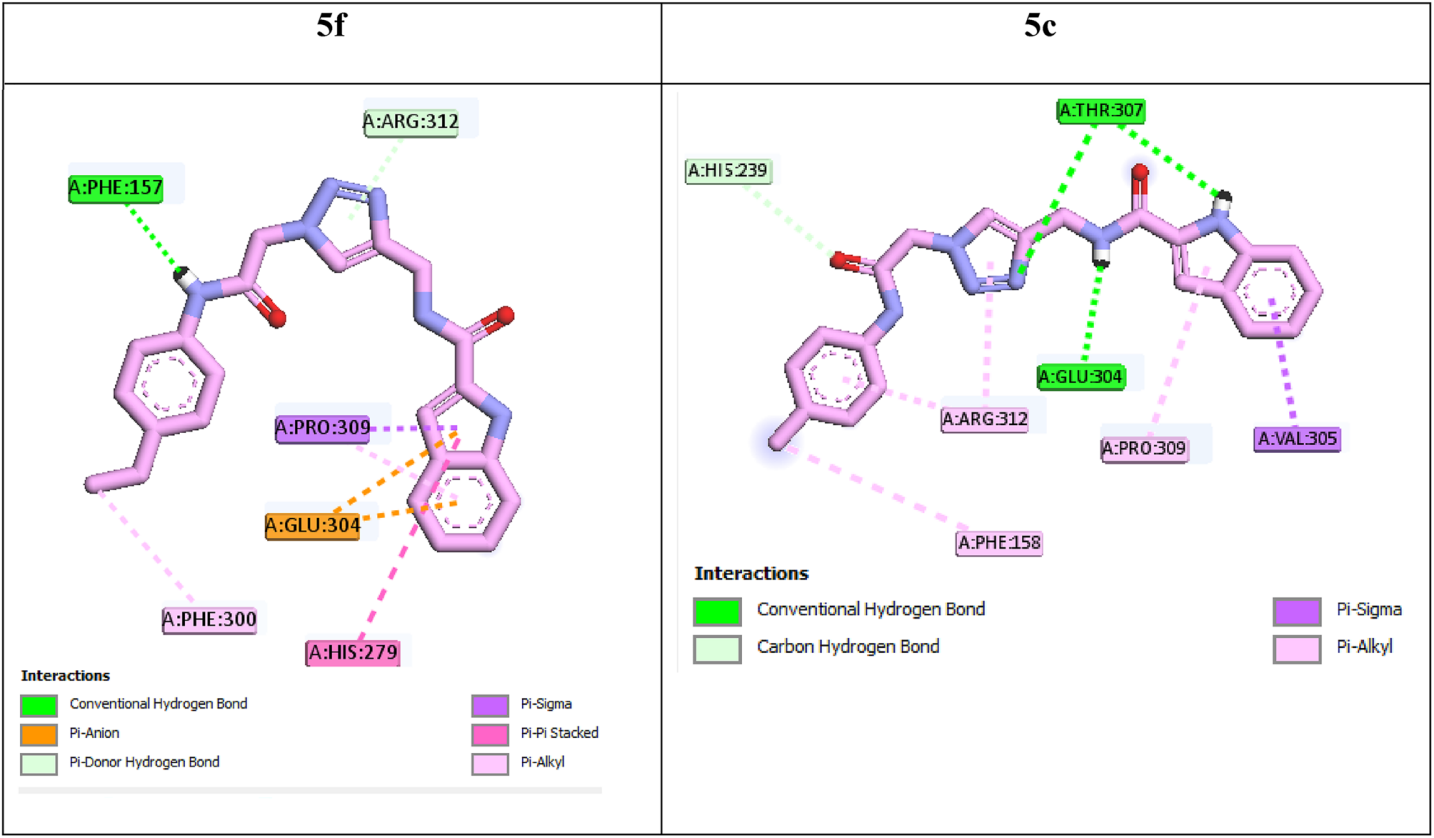
2D interaction modes of compounds **5f** and **5c** in the α-glucosidase active site.

**Figure 8:**
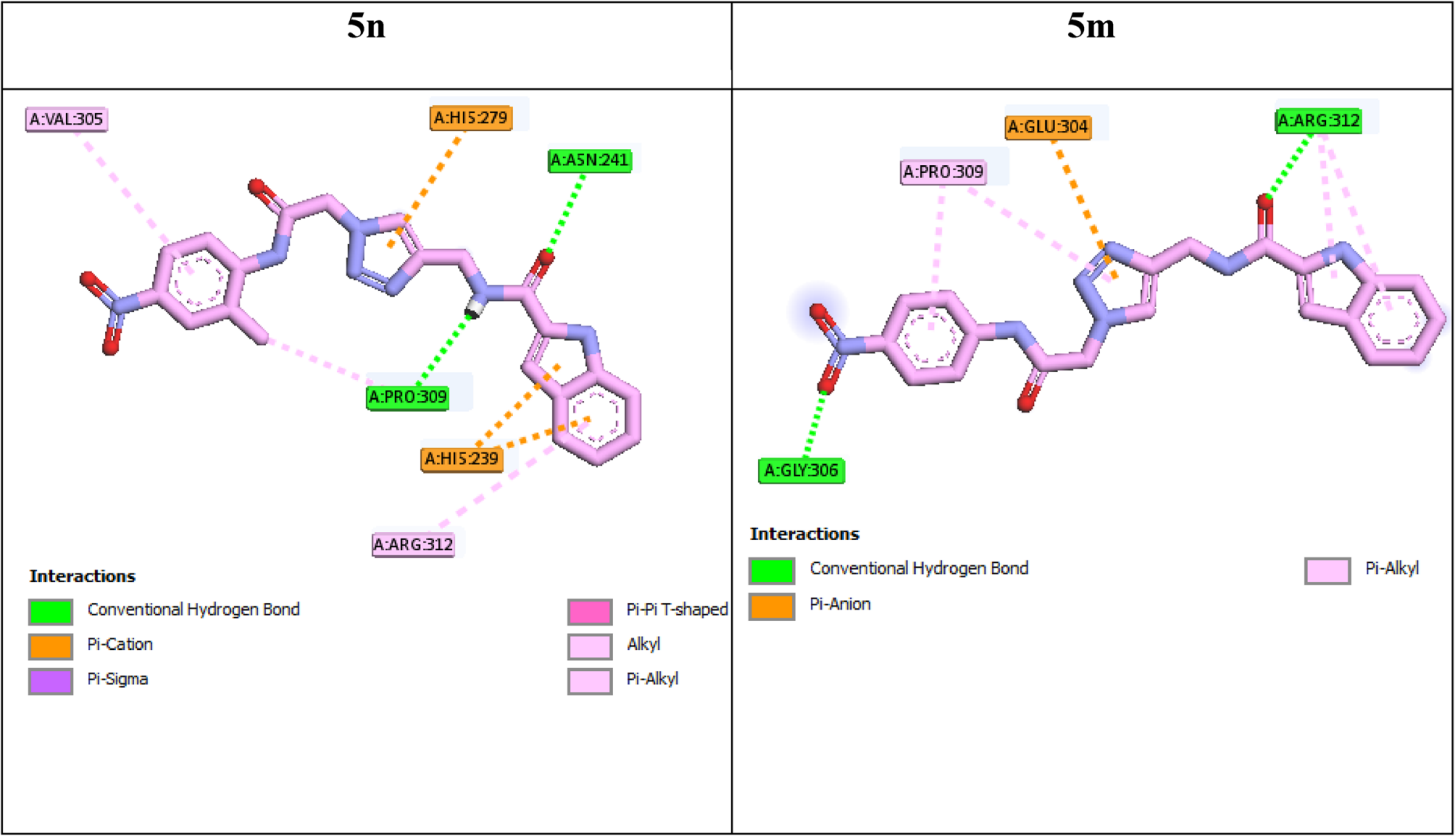
2D interaction modes of compounds **5n** and **5m** in the α-glucosidase active site.

Interaction modes of the selected compounds and acarbose were showed in the Figs. 6, 7 and 8. Acarbose as the standard α-glucosidase inhibitor with binding energy (BE) of − 4.04 kcal/mol established interactions with residues Asn241 (H-bond), Glu304 (H-bond), Ser308 (H-bond), Thr301 (H-bond), Thr307 (H-bond), Pro309 (H-bond), Arg312 (H-bond), Gln322 (H-bond), His279 (hydrophobic), Val305 (non-classical H-bond), His239 (non-classical H-bond), Thr307 (unfavorable), and Arg312 (unfavorable) in the active site of target enzyme^15^.

The most active compound **5k** established a hydrogen bond with Thr301 and two non-classical hydrogen bonds with His279 and Ser308 (Fig. 6). The latter amino acid also formed an unfavorable interaction with compound **5k**. Three π-interactions between compound **5k** and the active site residues were observed: a π-anion interaction with Glu304, a π-cation interaction with His239, and a π–π interaction with Phe157. Furthermore, compound **5k** formed several hydrophobic interactions with residues Val316, Val305, Pro309, and Arg312. BE of compound **5k** was − 8.49 kcal/mol.

The second potent compound **5j** established two hydrogen bonds with residues Ser308 and Thr307 and a non-classical hydrogen bond with Arg439 (Fig. 6). This compound formed two π-anion interactions with Glu304. Compound **5j** also established several hydrophobic interactions with residues Tyr313, Pro309, Phe311, and Arg312 and BE of this compound was -9.01 kcal/mol.

4-Ethyl derivative **5f** as the third potent compound created a classical hydrogen bond with residue Phe157 and a non-classical hydrogen bond with residue Arg312 (Fig. 7). Two π-anion interactions observed between compound **5f** and Glu304. The latter compound also formed hydrophobic interactions with residues Phe300 and Pro309. BE of compound **5f** was − 8.73 kcal/mol. Replacement of 4-ethyl substituent with 4-methyl substituent as in the case of compound **5c**, created a negligible decrease in the inhibition effect (Table 1). As can be seen in the interaction mode of compound **5c**, this compound with BE of − 8.18 kcal/mol established three hydrogen bonds with Thr307 (two H-bond) and Glu304, a non-classical hydrogen bond with residue His239, and several hydrophobic interactions with Phe158, Arg312, Pro309, and Val305. The comparison of the BEs of compound **5f** with **5c** showed that the third potent compound **5f** with BE value lower than the fourth potent compound **5c** attached to active site. This finding is in agreement with in vitro obtained data (Table 1).

As mentioned in SAR study (Sect. 2.2), the addition of a methyl group on position 2 on phenyl ring of *N*-phenylacetamide moiety of 4-nitro derivative **5m**, as in the case of compound **5n**, the inhibitory activity increased to about two fold. This observation was confirmed by docking study in two ways:

1. Compound **5n** interacted with a greater number of important amino acids (Val305, His279, His239, Asn241, Pro309, and Arg312) in the active site in comparison to compound **5m** (Gly306, Pr0309, Glu304, and Arg312).
2. BE of compound **5n** (BE = − 8.17 kcal/mol) was lower than compound **5m** (BE = − 8.08 kcal/mol).

### Molecular dynamics

It is difficult to grasp the interaction between a ligand and a receptor or assess the stability and flexibility of the resultant complex without resorting to molecular dynamic simulation, given that all atoms in the natural environment are in perpetual motion. To achieve this goal, the protein–ligand complex is confined within a box, after which water molecules and ions are introduced. The dynamics of the ligand-receptor complex are then simulated within this controlled environment. According to the in vitro studies, compound **5k** demonstrates the highest potential for inhibiting α-glucosidase. The stability and flexibility of the α-glucosidase-**5k** complex were examined by simulating its molecular dynamics in an explicit hydration environment. To provide a basis for comparison, a molecular dynamics simulation was conducted on the α-glucosidase-acarbose complex, which serves as a standard inhibitor. This study used a two-step simulation approach. Initially, a 10 ns simulation was conducted to assess the stability of ligands (**5k** and acarbose) at their binding site on α-glucosidase. Upon confirming the ligands' stability, the simulation duration was extended by another 10 ns to further understand the behavior of these compounds within the enzyme's active site. This extended simulation reaffirmed the stability of **5k** and acarbose at their binding site on α-glucosidase. In the subsequent analysis, various tools were utilized to examine the trajectory file from the molecular dynamic simulation.

**Figure 9:**
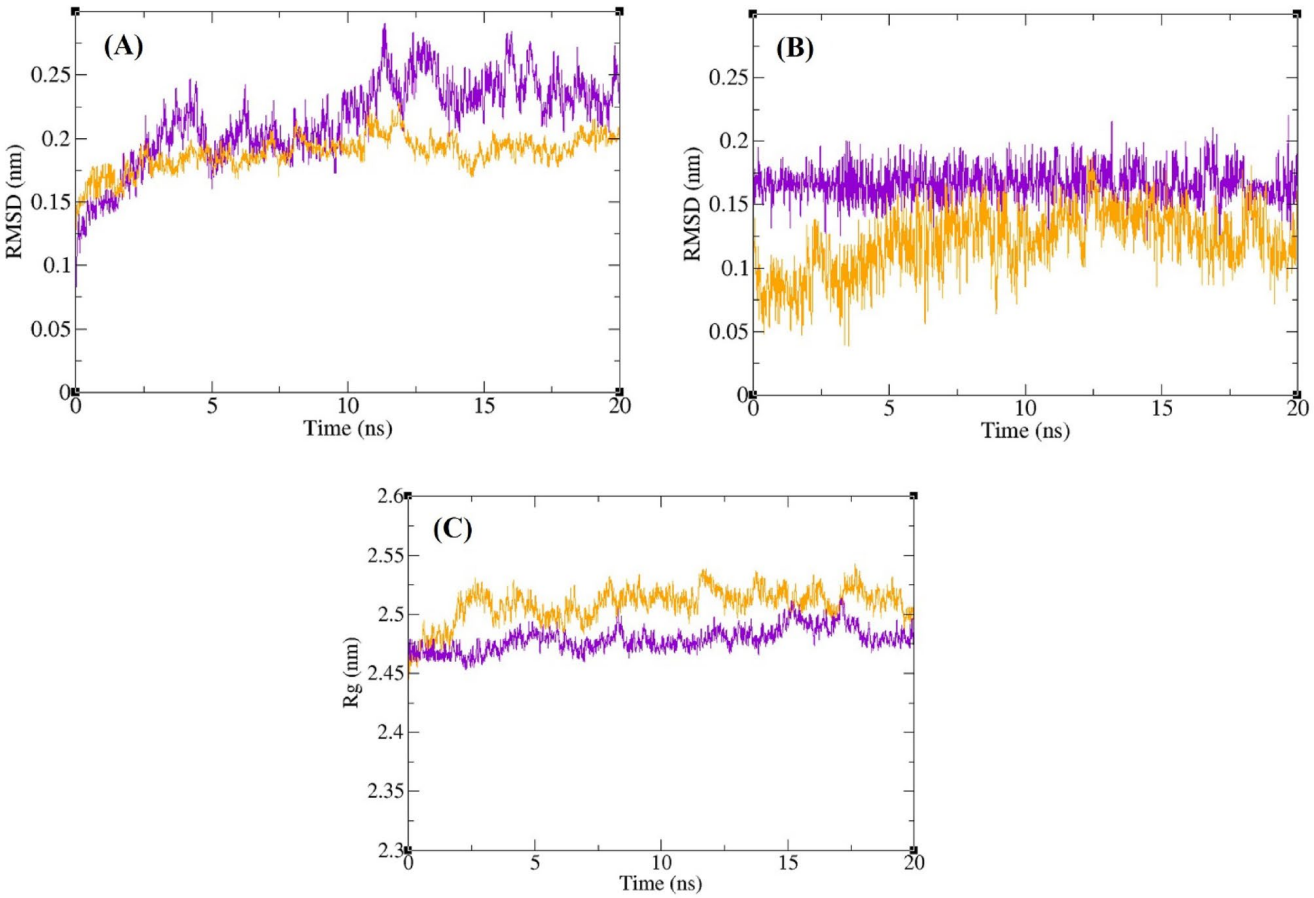
Superimposed RMSD of Cα atoms of α-glucosidase in complex with **5k** (violet) and acarbose (orange) (**A**). Superimposed RMSD of **5k** (violet) and acarbose (orange) in complex with α-glucosidase (**B**). Time dependence of the radius of gyration (Rg) graph of α-glucosidase in complex with **5k** (violet) and acarbose (orange) (**C**).

To measure the stability of the complexes, calculations of root-mean-square deviation (RMSD) and radius of gyration (Rg) were performed on all structures within the trajectory, and corresponding graphs were plotted. Additionally, to evaluate the residual flexibility of α-glucosidase and the flexibility of ligand atoms, the root mean square fluctuation (RMSF) of the backbone atoms in α-glucosidase and the heavy atoms in the ligands were computed. The results of the RMSD calculations are depicted in Fig. 9. According to this figure, the RMSD of the backbone atoms of α-glucosidase in complex with **5k** and acarbose exhibits minimal changes over time, never exceeding 0.3 nm. This indicates a stable protein structure. The average RMSD values for α-glucosidase in the complex with acarbose and/or **5k** were 0.18 nm and 0.21 nm, respectively. Similarly, the RMSD values of acarbose and/or **5k** in the complex with α-glucosidase remained consistently below 0.3 nm over time. The average RMSD values for acarbose and/or **5k** in complex with α-glucosidase were 0.12 nm and 0.16 nm, respectively. These observations collectively affirm the stability of both the enzyme and the ligands throughout the simulation period. The radius of gyration (Rg) of α-glucosidase was computed to assess protein compactness during the simulation, as depicted in Fig. 9. The average Rg of α-glucosidase was measured at 2.50 nm and 2.48 nm in the complexes with acarbose and **5k**, respectively. In the presence of both acarbose and **5k**, the Rg value of α-glucosidase remained within the narrow range of 2.38 to 2.54 nm, showing no significant upward or downward trend throughout the simulation period. This consistent Rg range indicates the stable compactness of α-glucosidase in these complexes.

**Figure 10:**
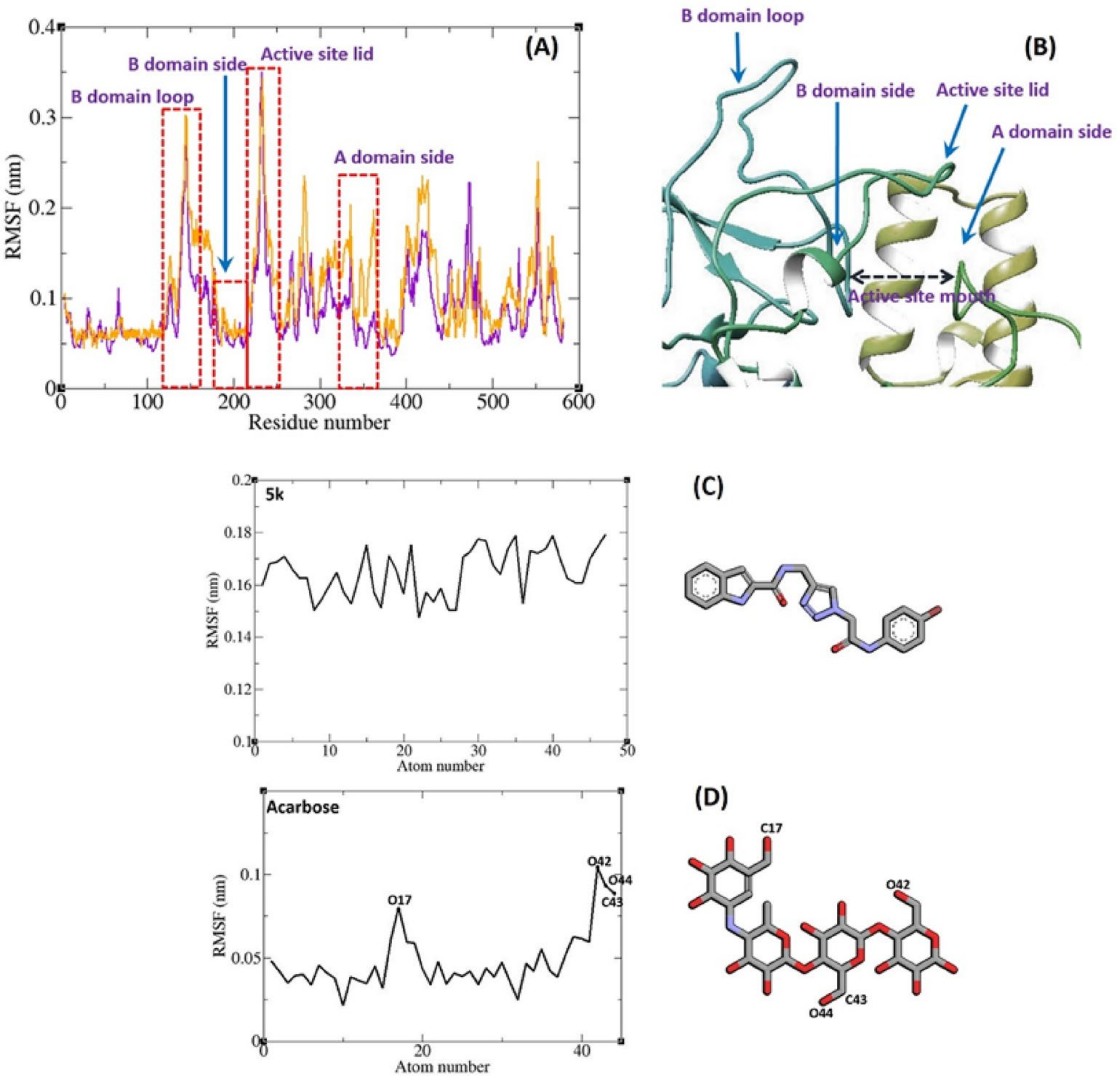
RMSF graph of the Cα atoms of α-glucosidase in complex with **5k** (violet) and acarbose (orange). Close-up representation of α-glucosidase active site (**B**). RMSF graph of the heavy atoms of **5k** (**C**) and acarbose (**D**) in complex with α-glucosidase. Structure of these compounds are illustrated.

Figure 10 illustrates the root mean square fluctuation (RMSF) of α-Glucosidase residues in complex with acarbose and **5k**. α-Glucosidase is comprised of various domains, each with distinct structures and functions. As depicted in Fig. 10, different parts of this protein exhibit dissimilar fluctuation patterns. Notably, the fluctuation of α-glucosidase residues in the complexes with acarbose and **5k** does not significantly differ; they follow the same pattern. Within α-glucosidase, there exists a cleft between the A domain and B domain, housing the enzyme's active site. Residues from these domains situated in this cleft, which contribute to non-bonded interactions with ligands, display lower fluctuations. This phenomenon aligns with the common observation in proteins, where loops typically exhibit the highest fluctuations. In α-glucosidase, the residues forming the B domain loop and the active site lid also demonstrate significant fluctuations. In Fig. 10, the fluctuation of heavy atoms in acarbose and **5k** is depicted. It is evident that the RMSF of all heavy atoms in these ligands is less than 2 nm. This minimal fluctuation serves as an indicator of their stable complex with α-glucosidase, suggesting that intermolecular interactions effectively limit their movements.

### Binding free energy analysis

**Figure 11:**
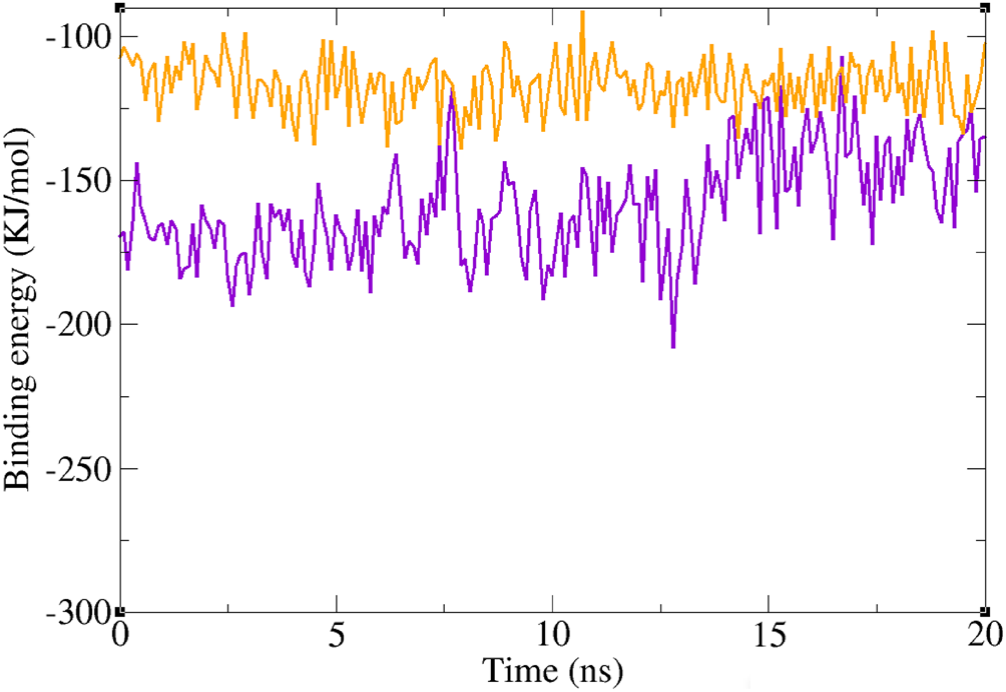
Diagram of binding energy changes during the last 20 ns of simulation time. α-glucosidase in complex with acarbose (orange), and **5k** (violet).

The assessment of ligand binding energy to a receptor can be conducted through the molecular mechanic/Poisson–Boltzmann surface area (MM/PBSA) method, providing insights into the predominant interactions within a ligand–receptor complex. Molecular docking, relying on singular structural snapshots, lacks precision compared to molecular dynamics (MD) simulations, which offer multiple snapshots over time, thus enhancing the accuracy of binding energy estimation. Results from the free binding energy analysis are delineated in Table 2. In this investigation, both acarbose and **5k** exhibited negative binding energies. Acarbose, a recognized inhibitor, demonstrated an average MM/PBSA free binding energy of − 115.7 kJ/mol with α-glucosidase, while **5k** manifested a binding free energy of − 159.4 kJ/mol. Figure 8 illustrates the binding energy changes over the 20 ns of MD simulation. Both complexes displayed consistent negative binding energies, indicative of stability. **5k** showed a binding energy even higher than that of acarbose, which indicates its high affinity to bind to α-glucosidase. Further investigation of free energy components revealed that the molecular mechanics interaction energy (van der Waals energy + electrostatic energy) favored complex formation, while solvation energy (polar solvation energy + solvent accessible surface area energy) impeded it (Fig. 11).

**Table 2:** Binding free energy (KJ/mol) for acarbose and **5k**.

Complex	Van der Waals energy	Electrostatic energy	Polar solvation energy	SASA^a^ energy	Binding energy
5k	− 237.6	− 35.5	154.5	− 40.8	− 159.4
Acarbose	− 219.9	− 30.1	163.8	− 29.5	− 115.7

^a^Solvent-accessible surface area.

**Figure 12:**
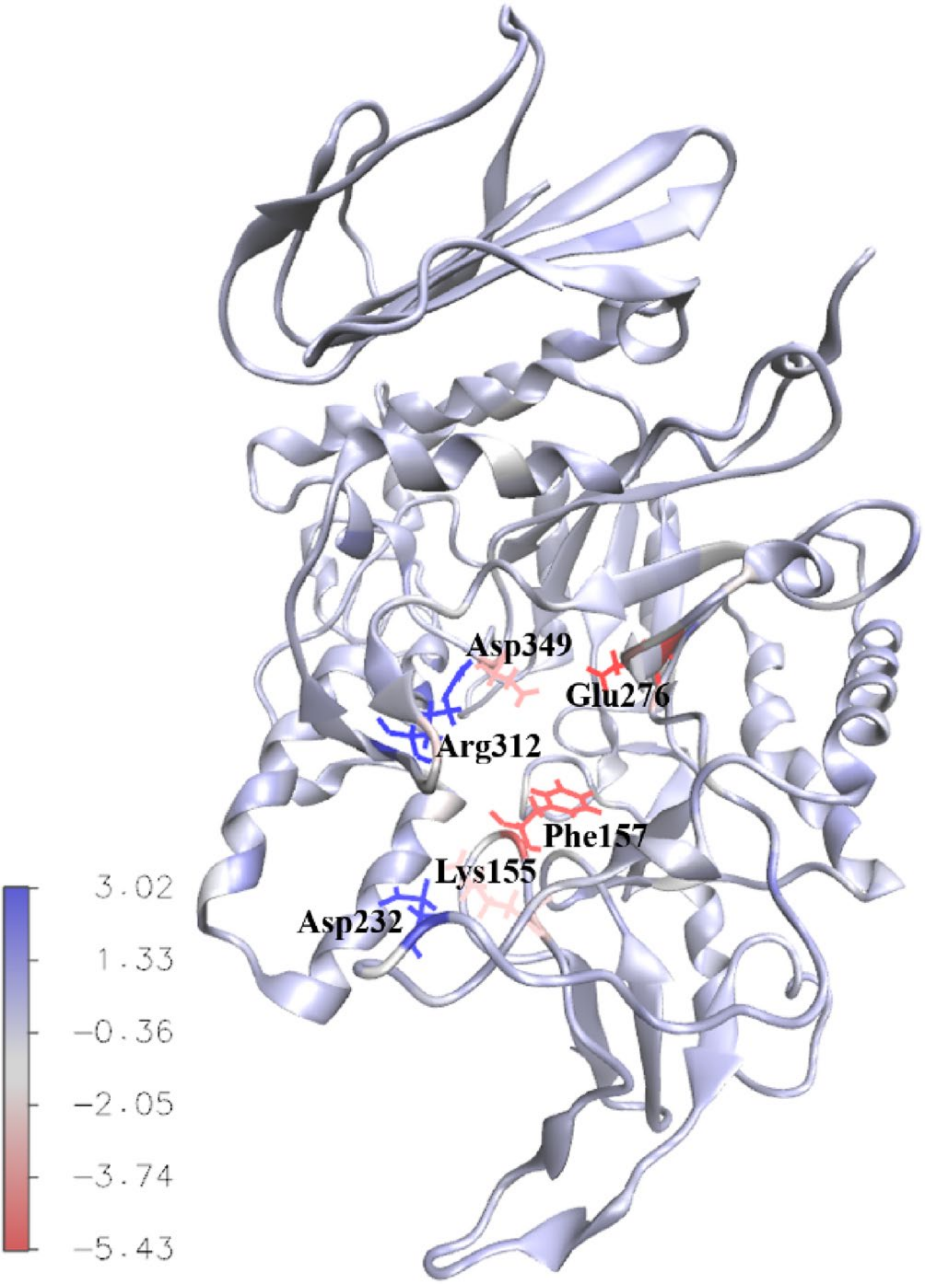
Residues with the largest and smallest contribution to the binding energy (KJ/mol) of α-glucosidase-**5k** complex.

**Figure 13:**
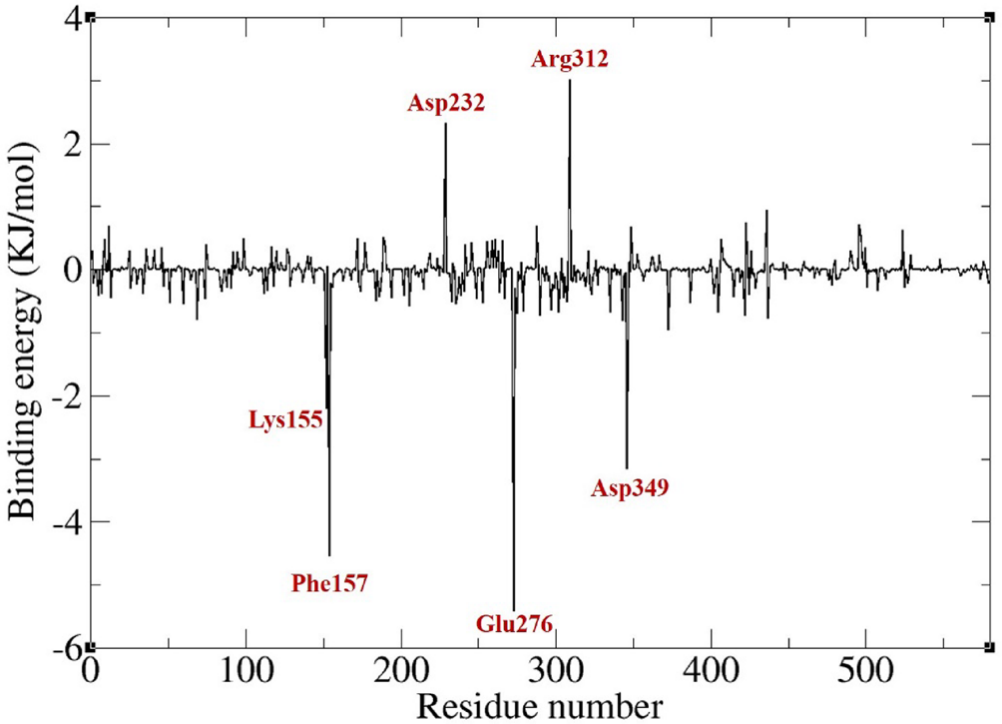
Contribution of α-glucosidase residues to the binding energy (KJ/mol) of α-glucosidase-**5k** complex.

The contribution of individual protein residues to binding energy were calculated too. Most residues deemed significant in ligand-receptor interaction via docking studies exhibited negative values in dynamic simulations, though some had minimal impact. Additionally, new residues with substantial binding energy contributions emerged, highlighting the dynamic nature of macromolecules and ligands, which can reveal previously unnoticed intermolecular interactions. Notably, four residues including Lys155, Phe157, Glu276 and Asp349 consistently played a significant role across all complexes with the most negative energies while Asp232 and Arg312 had the most positive energies (Figs. 12 and 13).

## Conclusion

In conclusion, we designed, synthesized, and screened highly potent α-glucosidase inhibitors from the connection of indole to *N*-phenylacetamide-1,2,3-triazole-carboxamide moiety. In this regard, 14 derivatives **5a–n** were synthesized by alteration of substituents on phenyl ring of *N*-phenylacetamide moiety. All the synthesized compounds **5a–n** were more potent than standard inhibitor and selected compound for their design. Moreover, SAR study demonstrated that 4-bromo derivative **5k** exhibited the best activity against the title enzyme. This compound showed 28 times better inhibitory effect than acarbose. Molecular modeling and molecular dynamics demonstrated that studied new compounds with acceptable binding energies interacted with important amino acids of the α-glucosidase active site. These data strongly correlated with the observed in vitro anti-α-glucosidase activity of our new compounds. Thus, presented new scaffold could be a good candidate for initiating a lead anti-diabetes drug discovery program.

## Experimental

### Synthesis of *N*-(prop-2-yn-1-yl)-1H-indole-2-carboxamide 3

To a mixture of 1*H*-indole-2-carboxylic acid **1** (10 mmol) and propargylamine **2** (10 mmol) in DMF (50 mL), TBTU (13 mmol) and Et_3_N (13 mmol) were added and the obtained mixture was stirred for 24 h at room temperature (RT). After completion of the reaction, water was added to this reaction mixture and the observed participate was filtered to give pure *N*-(prop-2-yn-1-yl)-1H-indole-2-carboxamide **3**.

### General procedure for the synthesis of *N*-phenylacetamide-1,2,3-triazole-indole-2-carboxamide derivatives 5a–n

In the next step, *N*-(prop-2-yn-1-yl)-1H-indole-2-carboxamide **3** (1 mmol), sodium ascorbate (15 mol %, 0.13 g), and CuSO_4_ (7 mol%) were added to a stirred mixture of the freshly prepared azide derivatives **4a–n** in H_2_O and t-BuOH (10 ml, 1:1) at RT. After 20–24 h, this reaction mixture was diluted with a mixture of ice and water, precipitated products **5a–n** were filtered off, washed with water, and purified by recrystallization in ethanol.

### *N*-((1-(2-oxo-2-(phenylamino)ethyl)-1*H*-1,2,3-triazol-4-yl)methyl)-1*H*-indole-2-carboxamide (5a)

Yield: 75%. Light brown solid. M.p. 216–218 °C. ^1^H NMR (301 MHz, DMSO-*d*_6_) δ 11.65 (s, 1H), 10.48 (s, 1H), 9.11 (s, 1H), 8.07 (s, 1H), 7.80–6.86 (m, 10H), 5.34 (s, 2H), 4.78–4.44 (m, 2H). ^13^C NMR (76 MHz, DMSO-*d*_6_) δ 164.70, 161.55, 138.89, 136.93, 131.94, 129.38, 127.55, 125.17, 124.22, 123.81, 122.00, 120.19, 120.16, 119.66, 112.78, 103.23, 52.65, 34.87. *Anal*. Calcd for C_20_H_18_N_6_O_2_: C, 64.16; H, 4.85; N, 22.45; Found: C 63.91; H 5.02; N 22.29.

### *N-*((1-(2-oxo-2-(m-tolylamino)ethyl)-1*H*-1,2,3-triazol-4-yl)methyl)-1*H*-indole-2-carboxamide (5b)

Yield: 81%. Light brown solid. M.p. 224–226 °C. ^1^H NMR (301 MHz, DMSO-*d*_6_) δ 11.68 (s, 1H), 10.43 (s, 1H), 9.15 (d, *J* = 5.6 Hz, 1H), 8.09 (s, 1H), 7.65 (d, *J* = 7.8 Hz, 1H), 7.57–7.35 (m, 3H), 7.22 (d, *J* = 6.6 Hz, 3H), 7.06 (t, *J* = 7.4 Hz, 1H), 6.92 (d, *J* = 7.4 Hz, 1H), 5.36 (s, 2H), 4.83–4.44 (m, 2H), 2.29 (s, 3H). ^13^C NMR (76 MHz, DMSO-*d*_6_) δ 164.65, 161.61, 138.83, 138.62, 136.97, 131.97, 129.21, 127.59, 125.25, 124.96, 123.84, 122.02, 120.24, 120.21, 116.89, 112.81, 103.29, 52.71, 34.90, 21.63. *Anal*. Calcd for C_21_H_20_N_6_O_2_: C, 64.94; H, 5.19; N, 21.64; Found: C 64.80; H 5.41; N 21.76.

### *N*-((1-(2-oxo-2-(*p*-tolylamino)ethyl)-1*H*-1,2,3-triazol-4-yl)methyl)-1*H*-indole-2-carboxamide (5c)

Yield: 81%. Light brown solid. M.p. 231–233 °C. ^1^H NMR (301 MHz, DMSO-*d*_6_) δ 11.64 (s, 1H), 10.39 (s, 1H), 9.11 (d, *J* = 5.7 Hz, 1H), 8.04 (s, 1H), 7.75–7.32 (m, 4H), 7.35–6.89 (m, 5H), 5.31 (s, 2H), 4.60 (d, *J* = 5.4 Hz, 2H), 2.26 (s, 3H). ^13^C NMR (76 MHz, DMSO-*d*_6_) δ 164.44, 161.53, 136.92, 136.38, 133.19, 131.93, 129.75, 127.54, 125.13, 123.80, 122.00, 120.19, 119.75, 119.65, 112.78, 103.21, 52.61, 34.85, 20.92. *Anal*. Calcd for C_21_H_20_N_6_O_2_: C, 64.94; H, 5.19; N, 21.64; Found: C 64.77; H 4.98; N 21.45.

### *N*-((1-(2-((2,3-dimethylphenyl)amino)-2-oxoethyl)-1*H*-1,2,3-triazol-4-yl)methyl)-1H-indole-2-carboxamide (5d)

Yield: 83%. Light brown solid. M.p. 205–207 °C. ^1^H NMR (301 MHz, DMSO-*d*_6_) δ 11.66 (s, 1H), 9.87 (s, 1H), 9.12 (s, 1H), 8.06 (s, 1H), 7.78–6.77 (m, 8H), 5.39 (s, 2H), 4.62 (s, 2H), 2.26 (s, 3H), 2.12 (s, 3H). ^13^C NMR (76 MHz, DMSO-*d*_6_) δ 164.95, 161.57, 137.61, 136.95, 135.73, 131.95, 131.50, 127.74, 127.56, 125.77, 125.15, 123.81, 123.72, 122.00, 120.23, 120.20, 112.79, 103.25, 52.36, 34.89, 20.60, 14.48. *Anal*. Calcd for C_22_H_22_N_6_O_2_: C, 65.66; H, 5.51; N, 20.88; Found: C 65.38; H 5.37; N 20.72.

### *N*-((1-(2-((2,6-dimethylphenyl)amino)-2-oxoethyl)-1*H*-1,2,3-triazol-4-yl)methyl)-1*H*-indole-2-carboxamide (5e)

Yield: 85%. Light brown solid. M.p. 208–210 °C. ^1^H NMR (301 MHz, DMSO-*d*_6_) δ 11.64 (s, 1H), 9.77 (s, 1H), 9.11 (d, *J* = 5.8 Hz, 1H), 8.05 (s, 1H), 7.63 (d, *J* = 7.7 Hz, 1H), 7.46 (d, *J* = 8.0 Hz, 1H), 7.31–6.92 (m, 6H), 5.38 (s, 2H), 4.70–4.45 (m, 2H), 2.17 (s, 6H). ^13^C NMR (76 MHz, DMSO-*d*_6_) δ 164.55, 161.55, 136.93, 135.55, 134.68, 131.94, 128.22, 127.54, 127.22, 124.98, 123.81, 122.00, 120.21, 120.20, 112.78, 103.22, 52.04, 34.87, 18.50. *Anal*. Calcd for C_22_H_22_N_6_O_2_: C, 65.66; H, 5.51; N, 20.88; Found: C 65.41; H 5.74; N 20.98.

### *N*-((1-(2-((4-ethylphenyl)amino)-2-oxoethyl)-1*H*-1,2,3-triazol-4-yl)methyl)-1*H*-indole-2-carboxamide (5f)

Yield: 82%. Light brown solid. M.p. 203–205 °C. ^1^H NMR (301 MHz, DMSO-*d*_6_) δ 11.65 (s, 1H), 10.41 (s, 1H), 9.12 (s, 1H), 8.06 (s, 1H), 7.71–7.38 (m, 4H), 7.31–7.00 (m, 5H), 5.32 (s, 2H), 4.62 (s, 2H), 2.71–2.37 (m, 2H), 1.16 (t, *J* = 7.2 Hz, 3H). ^13^C NMR (76 MHz, DMSO-*d*_6_) δ 164.44, 161.55, 139.64, 136.93, 136.57, 131.94, 128.56, 127.55, 125.07, 123.81, 122.00, 120.20, 119.84, 119.74, 112.78, 103.23, 52.63, 34.87, 28.06, 16.11. *Anal*. Calcd for C_22_H_22_N_6_O_2_: C, 65.66; H, 5.51; N, 20.88; Found: C 65.43; H 5.32; N 21.01.

### *N*-((1-(2-((4-methoxyphenyl)amino)-2-oxoethyl)-1*H*-1,2,3-triazol-4-yl)methyl)-1*H*-indole-2-carboxamide (5g)

Yield: 81%. Light brown solid. M.p. 209–211 °C. ^1^H NMR (301 MHz, DMSO-*d*_6_) δ 11.66 (s, 1H), 10.37 (s, 1H), 9.12 (s, 1H), 8.07 (s, 1H), 7.80–7.33 (m, 4H), 7.32–6.82 (m, 5H), 5.31 (s, 2H), 4.62 (s, 2H), 3.73 (s, 3H). ^13^C NMR (76 MHz, DMSO-*d*_6_) δ 164.17, 161.56, 155.98, 136.94, 131.98, 131.94, 127.55, 125.20, 123.82, 122.01, 121.32, 121.23, 120.21, 114.47, 112.78, 103.25, 55.61, 52.60, 34.87. *Anal*. Calcd for C_21_H_20_N_6_O_3_: C, 62.37; H, 4.98; N, 20.78; Found: C 62.14; H 5.21; N 20.63.

### *N*-((1-(2-((3-chlorophenyl)amino)-2-oxoethyl)-1*H*-1,2,3-triazol-4-yl)methyl)-1*H*-indole-2-carboxamide (5h)

Yield: 70%. Light brown solid. M.p. 211–213 °C. ^1^H NMR (301 MHz, DMSO-*d*_6_) δ 11.64 (s, 1H), 10.07 (s, 1H), 9.11 (s, 1H), 8.06 (s, 1H), 7.77–7.02 (m, 9H), 5.44 (s, 2H), 4.60 (s, 2H). ^13^C NMR (76 MHz, DMSO-*d*_6_) δ 165.41, 161.53, 136.92, 134.62, 131.92, 130.09, 128.03, 127.53, 127.16, 126.69, 126.30, 124.95, 123.80, 121.99, 120.21, 120.19, 112.77, 103.20, 52.36, 34.84. *Anal*. Calcd for C_20_H_17_ClN_6_O_2_: C, 58.76; H, 4.19; N, 20.56; Found: C 58.54; H 3.98; N 20.69.

### *N*-((1-(2-((4-chlorophenyl)amino)-2-oxoethyl)-1*H*-1,2,3-triazol-4-yl)methyl)-1*H*-indole-2-carboxamide (5i)

Yield: 74%. Light brown solid. M.p. 225–227 °C. ^1^H NMR (301 MHz, DMSO-*d*_6_) δ 11.64 (s, 1H), 10.63 (s, 1H), 9.12 (s, 1H), 8.06 (s, 1H), 7.76–6.84 (m, 9H), 5.34 (s, 2H), 4.61 (s, 2H).

^13^C NMR (76 MHz, DMSO-*d*_6_) δ 164.91, 161.55, 137.83, 136.92, 131.92, 129.30, 127.82, 127.54, 125.23, 123.81, 121.99, 121.33, 121.23, 120.20, 112.78, 103.23, 52.63, 34.86.

*Anal*. Calcd for C_20_H_17_ClN_6_O_2_: C, 58.76; H, 4.19; N, 20.56; Found: C 58.59; H 4.37; N 20.33.

### *N*-((1-(2-((2,4-dichlorophenyl)amino)-2-oxoethyl)-1*H*-1,2,3-triazol-4-yl)methyl)-1*H*-indole-2-carboxamide (5j)

Yield: 74%, Light brown solid. M.p. 233–235 °C. ^1^H NMR (301 MHz, DMSO-*d*_6_) δ 11.66 (s, 1H), 10.17 (s, 1H), 9.14 (s, 1H), 8.38–6.71 (m, 10H), 5.48 (s, 2H), 4.64 (s, 2H).

^13^C NMR (76 MHz, DMSO-*d*_6_) δ 165.59, 161.60, 136.95, 133.84, 131.92, 130.22, 129.52, 128.14, 127.56, 127.48, 127.17, 125.12, 123.84, 122.01, 120.26, 120.22, 112.80, 103.31, 52.45, 34.91.

*Anal*. Calcd for C_20_H_16_Cl_2_N_6_O_2_: C, 54.19; H, 3.64; N, 18.96; Found: C 53.96; H 3.82; N 19.18.

### *N*-((1-(2-((4-bromophenyl)amino)-2-oxoethyl)-1*H*-1,2,3-triazol-4-yl)methyl)-1*H*-indole-2-carboxamide (5k)

Yield: 81%. Light brown solid. M.p. 236–238 °C. ^1^H NMR (301 MHz, DMSO-*d*_6_) *δ* 11.63 (s, 1H), 10.63 (s, 1H), 9.11 (s, 1H), 8.11–7.04 (m, 10H), 5.34 (s, 2H), 4.60 (s, 2H). ^13^C NMR (76 MHz, DMSO-*d*_6_) *δ* 164.88, 161.55, 138.23, 136.92, 132.21, 131.90, 127.53, 125.29, 123.82, 121.99, 121.71, 121.62, 120.21, 115.87, 112.78, 103.25, 52.76, 34.91. *Anal*. Calcd for C_20_H_17_BrN_6_O_2_: C, 52.99; H, 3.78; N, 18.54; Found: C 52.79; H 4.01; N 18.31.

### *N*-((1-(2-((3-nitrophenyl)amino)-2-oxoethyl)-1*H*-1,2,3-triazol-4-yl)methyl)-1*H*-indole-2-carboxamide (5l)

Yield: 79%. Brown solid. M.p. 254–256 °C. ^1^H NMR (301 MHz, DMSO-*d*_6_) δ 11.65 (s, 1H), 10.99 (s, 1H), 9.12 (t, *J* = 5.6 Hz, 1H), 8.61 (s, 1H), 8.09 (s, 1H), 8.01–7.84 (m, 2H), 7.65 (t, *J* = 7.9 Hz, 2H), 7.47 (d, *J* = 8.2 Hz, 1H), 7.13 (dt, *J* = 44.7, 6.9 Hz, 3H), 5.41 (s, 2H), 4.63 (d, *J* = 5.6 Hz, 2H). ^13^C NMR (76 MHz, DMSO-*d*_6_) δ 165.63, 161.58, 148.44, 139.97, 136.94, 131.93, 130.87, 127.56, 125.66, 125.15, 123.82, 122.00, 121.83, 120.20, 118.77, 113.85, 112.78, 103.26, 52.65, 34.88. *Anal*. Calcd for C_20_H_17_N_7_O_4_: C, 57.28; H, 4.09; N, 23.38; Found: C 56.99; H 4.31; N 23.59.

### *N*-((1-(2-((4-nitrophenyl)amino)-2-oxoethyl)-1*H*-1,2,3-triazol-4-yl)methyl)-1*H*-indole-2-carboxamide (5m)

Yield: 69%. Brown solid. M.p. 259–261 °C. ^1^H NMR (301 MHz, DMSO-*d*_6_) δ 11.64 (s, 1H), 11.09 (s, 1H), 9.12 (s, 1H), 8.43–7.71 (m, 5H), 7.62 (d, *J* = 7.7 Hz, 1H), 7.45 (d, *J* = 8.0 Hz, 1H), 7.12 (d, *J* = 44.2 Hz, 3H), 5.43 (s, 2H), 4.61 (s, 2H). ^13^C NMR (76 MHz, DMSO-*d*_6_) δ 165.87, 161.55, 144.98, 143.03, 136.92, 131.91, 127.53, 125.58, 125.21, 123.82, 121.99, 120.20, 119.52, 119.48, 112.78, 103.23, 52.75, 34.86. *Anal*. Calcd for C_20_H_17_N_7_O_4_: C, 57.28; H, 4.09; N, 23.38; Found: C 57.02; H 4.28; N 23.17.

### *N*-((1-(2-((2-methyl-4-nitrophenyl)amino)-2-oxoethyl)-1*H*-1,2,3-triazol-4-yl)methyl)-1*H*-indole-2-carboxamide (5n)

Yield: 73%. Brown solid. M.p. 229–231 °C. ^1^H NMR (301 MHz, DMSO-*d*_6_) δ 11.63 (s, 1H), 10.04 (s, 1H), 9.11 (s, 1H), 8.35–7.79 (m, 5H), 7.62 (d, *J* = 7.6 Hz, 1H), 7.45 (d, *J* = 7.9 Hz, 1H), 7.27–6.93 (m, 3H), 5.49 (s, 2H), 4.61 (s, 2H), 2.41 (s, 3H). ^13^C NMR (76 MHz, DMSO-*d*_6_) δ 165.77, 161.54, 143.91, 142.59, 136.92, 131.92, 131.73, 127.53, 126.00, 124.82, 123.81, 123.67, 122.32, 121.99, 120.21, 120.19, 112.77, 103.22, 52.61, 34.88, 18.30. *Anal*. Calcd for C_21_H_19_N_7_O_4_: C, 58.19; H, 4.42; N, 22.62; Found: C 58.01; H 4.66; N 22.35.

### In vitro α-glucosidase and α-amylase inhibition assays

In vitro assays (inhibition effects and kinetics) of the new compounds **5a–n** performed according to pervious reported work^15^ (Supplementary Information S1).

### Docking study

Molecular modeling of the selected compounds **5c**, **5f**, **5j**, and **5k**, and molecular dynamics of the most potent compound **5k** done on modeled α-glucosidase based on our pervious reported works^15,20^. Coordinates of active site of the modeled α-glucosidase was placed as follows:

Center of the grid box: x = 12.5825, y = − 7.8955, and z = 12.519 Å

Dimensions of the active site box: 40 × 40 × 40 Å.

### Free binding energy calculations

The evaluation of the binding free energy of the protein–ligand complex was conducted utilizing the g_mmpbsa program^21–23^. Developed specifically for this purpose, g_mmpbsa facilitates the computation of the various constituents contributing to the binding free energy employing the molecular mechanics/Poisson-Boltzmann surface area (MM/PBSA) methodology. This computational tool enables the determination of the binding energy components pertinent to the protein–ligand complex which can be described as:

$$\begin{gathered} {\text{Free binding energy }} = {\text{ molecular mechanics interaction energy }}\left( {{\text{MMIE}}} \right) \, + {\text{solvation energy }}\left( {{\text{SE}}} \right) \hfill \\ {\text{MMIE }} = {\text{ van der Waals energy }} + {\text{Electrostatic energy}} \hfill \\ {\text{SE }} = {\text{ polar solvation energy }}\left( {{\text{PSE}}} \right) \, + {\text{nonpolar solvation energy }}\left( {\text{SASA energy}} \right) \hfill \\ {\text{PSE }} = {\text{ PSE}}_{{{\text{complex}}}} - \, \left( {{\text{PSE}}_{{{\text{protein}}}} + {\text{PSE}}_{{{\text{ligand}}}} } \right) \hfill \\ {\text{SASA}}_{{{\text{energy}}}} = {\text{ SASA}}_{{{\text{complex}}}} {-} \, \left( {{\text{SASA}}_{{{\text{protein}}}} + {\text{SASA}}_{{{\text{ligand}}}} } \right) \hfill \\ \end{gathered}$$

### Supplementary Information

Supplementary Information.

## Data Availability

The datasets used or analyzed during the current study are available from the corresponding author on reasonable request.
