# Supplementary material for: Design, synthesis, in vitro, and in silico anti-α-glucosidase assays of N-phenylacetamide-1,2,3-triazole-indole-2-carboxamide derivatives as new anti-diabetic agents
Source: Sci Rep. 2024 Jul 9;14:15791. doi: 10.1038/s41598-024-66201-y (PMC11233587; doi:10.1038/s41598-024-66201-y)

**Support information**

*N*-((1-(2-oxo-2-(phenylamino)ethyl)-1*H*-1,2,3-triazol-4-yl)methyl)-1*H*-indole-2-carboxamide (**5a**)

*
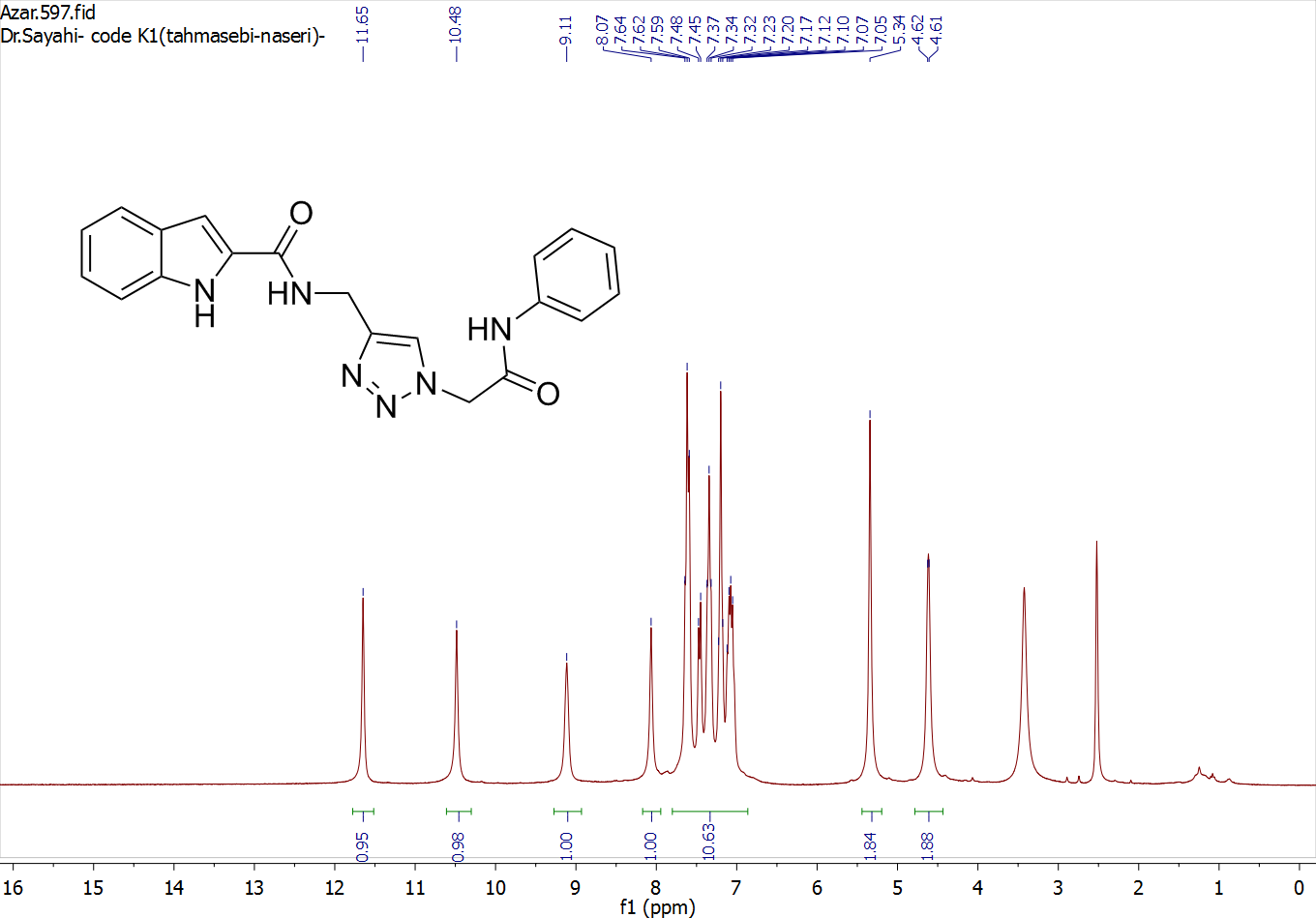
*

*
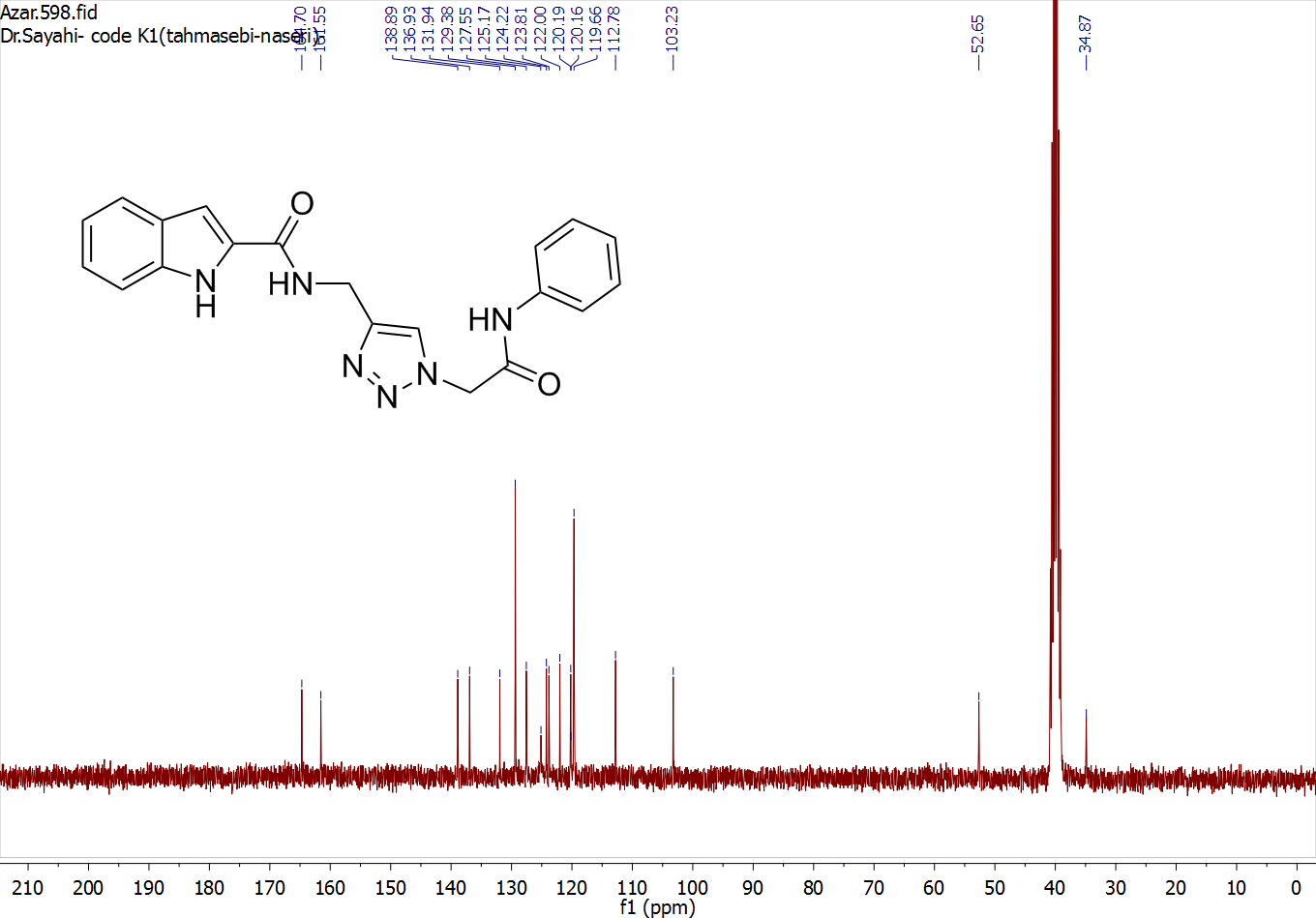
*

*N-*((1-(2-oxo-2-(m-tolylamino)ethyl)-1*H*-1,2,3-triazol-4-yl)methyl)-1*H*-indole-2-carboxamide (**5b**)

*
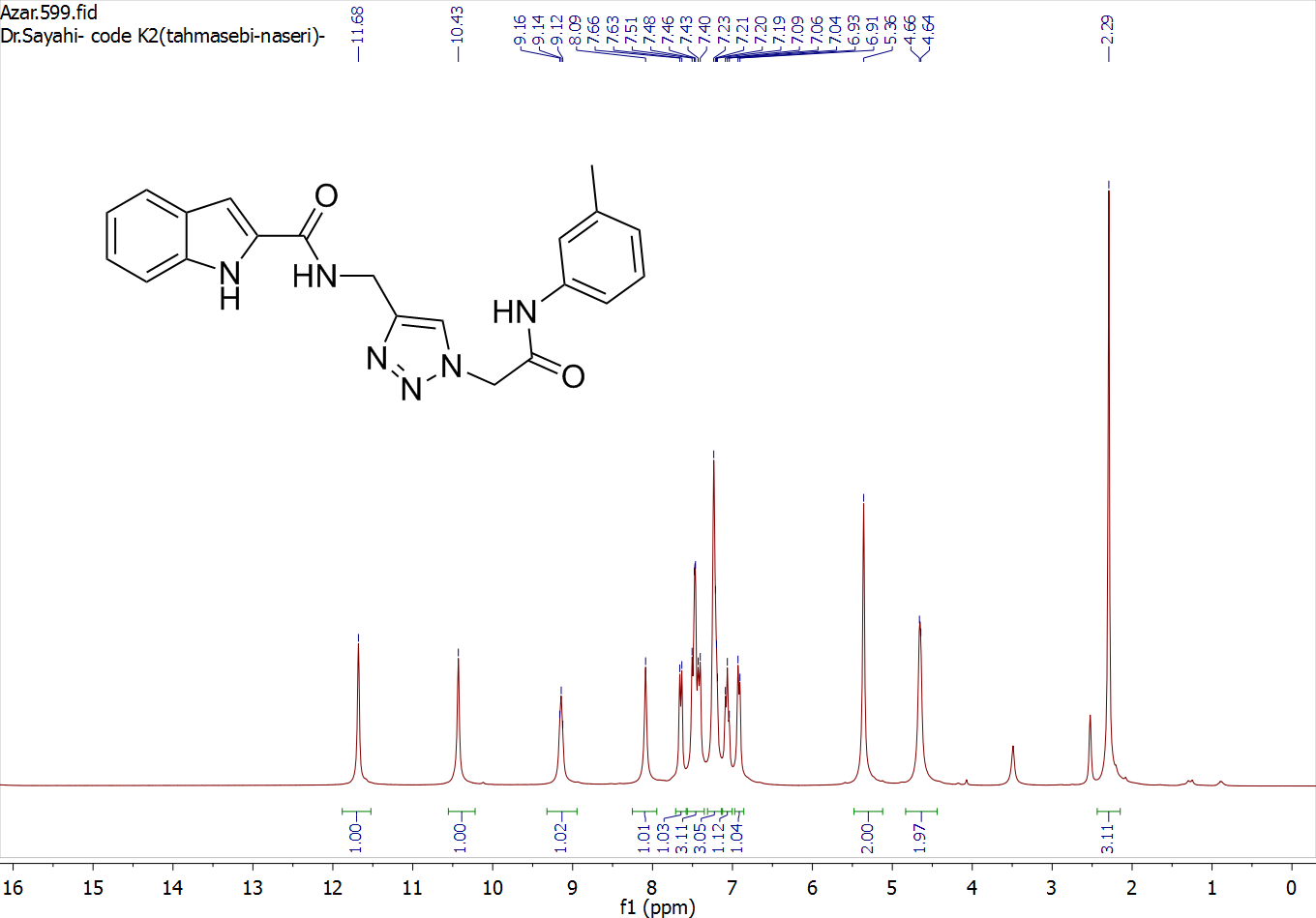
*

*
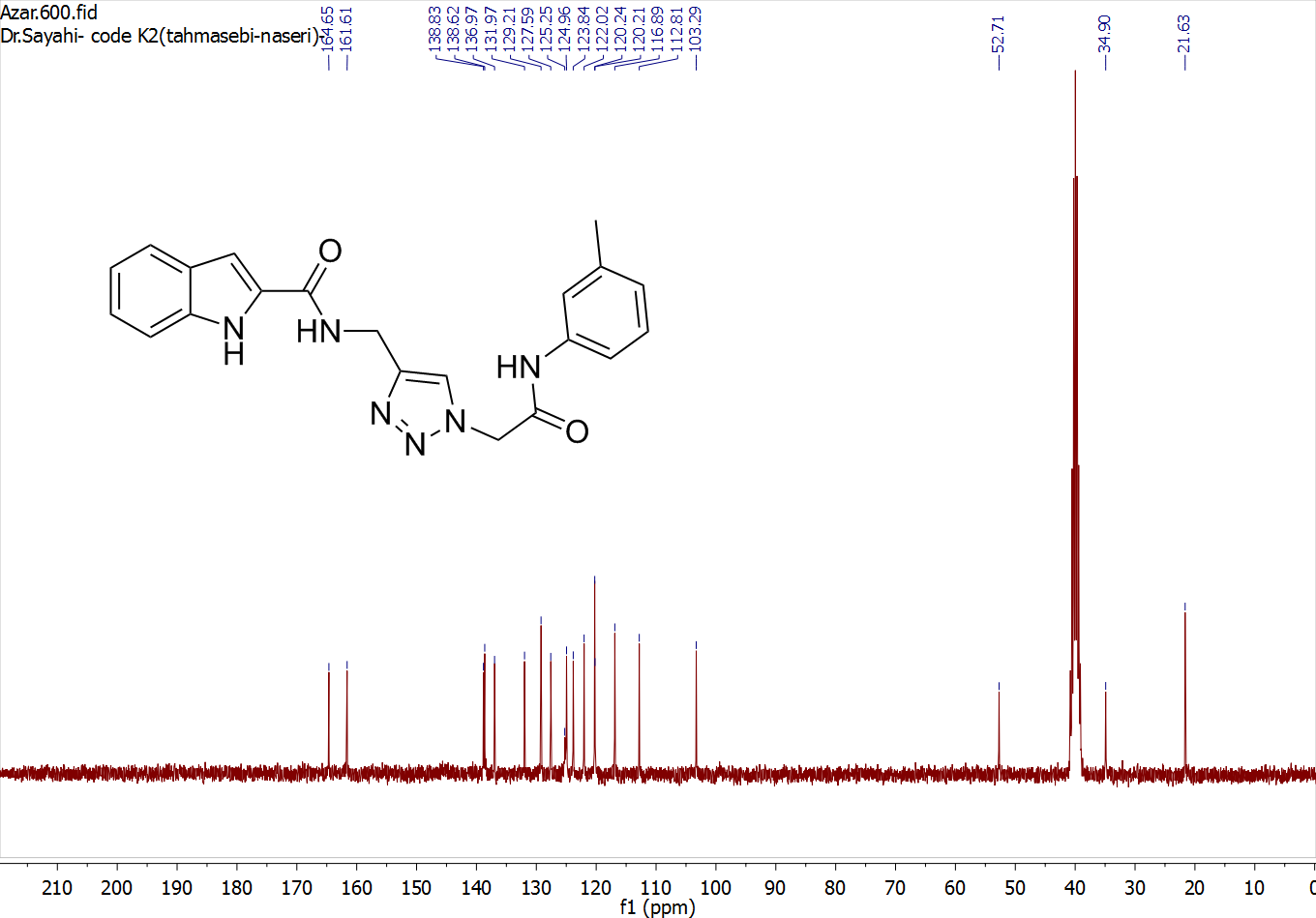
*

***N*-((1-(2-((4-bromophenyl)amino)-2-oxoethyl)-1*H*-1,2,3-triazol-4-yl)methyl)-1*H*-indole-2-carboxamide**

***
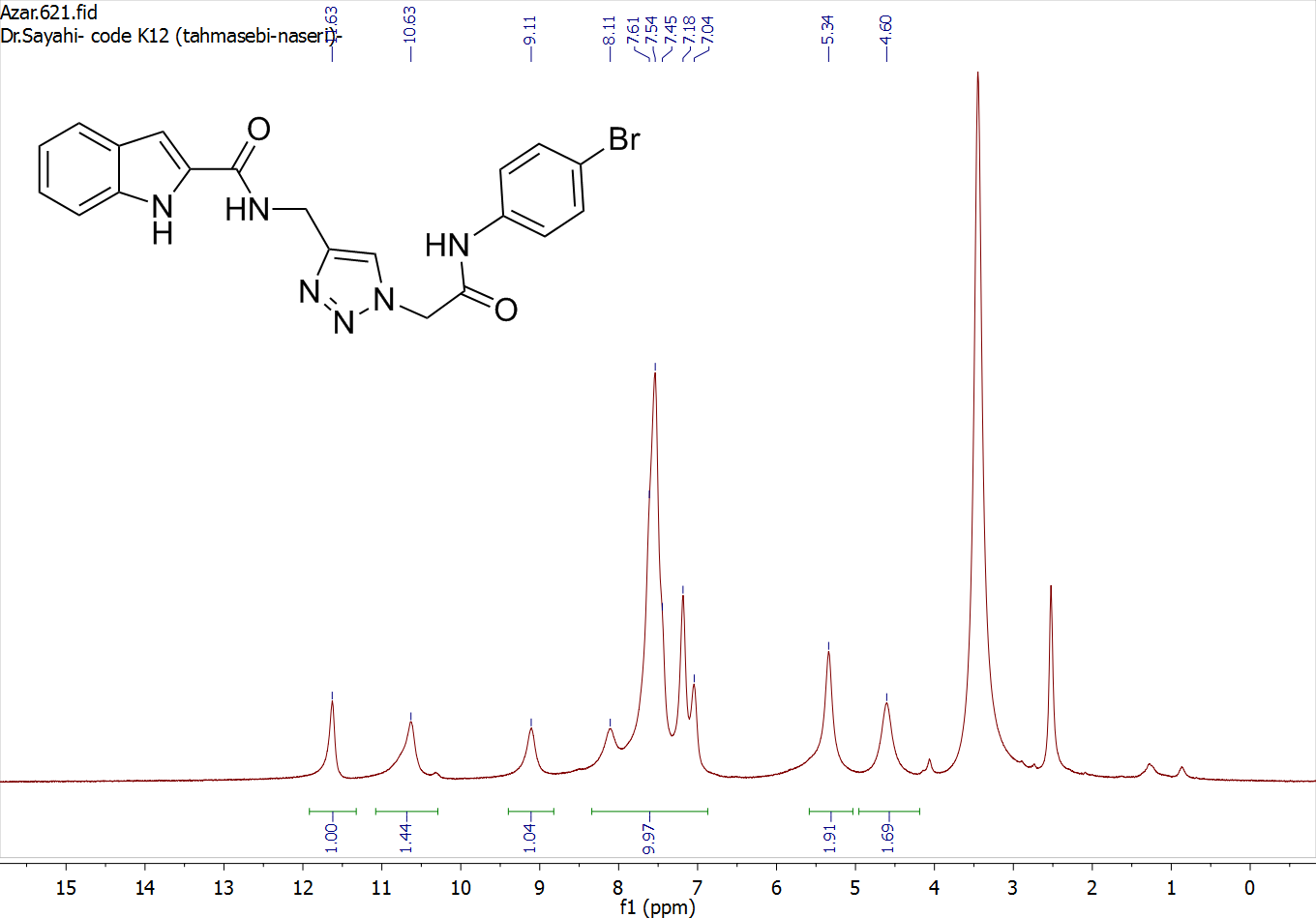
***

***
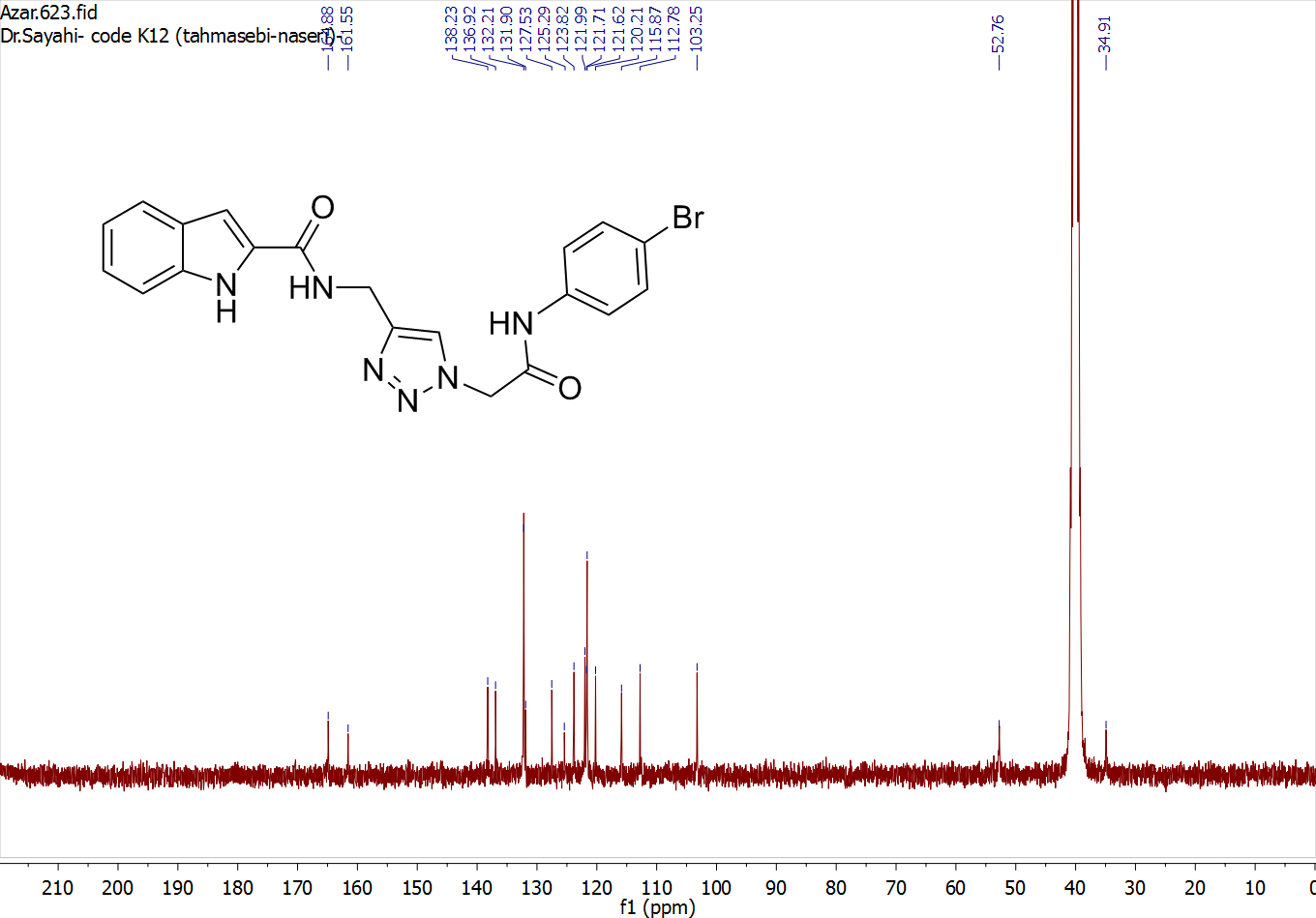
***

*N*-((1-(2-((3-nitrophenyl)amino)-2-oxoethyl)-1*H*-1,2,3-triazol-4-yl)methyl)-1*H*-indole-2-carboxamide (**5l**)

*
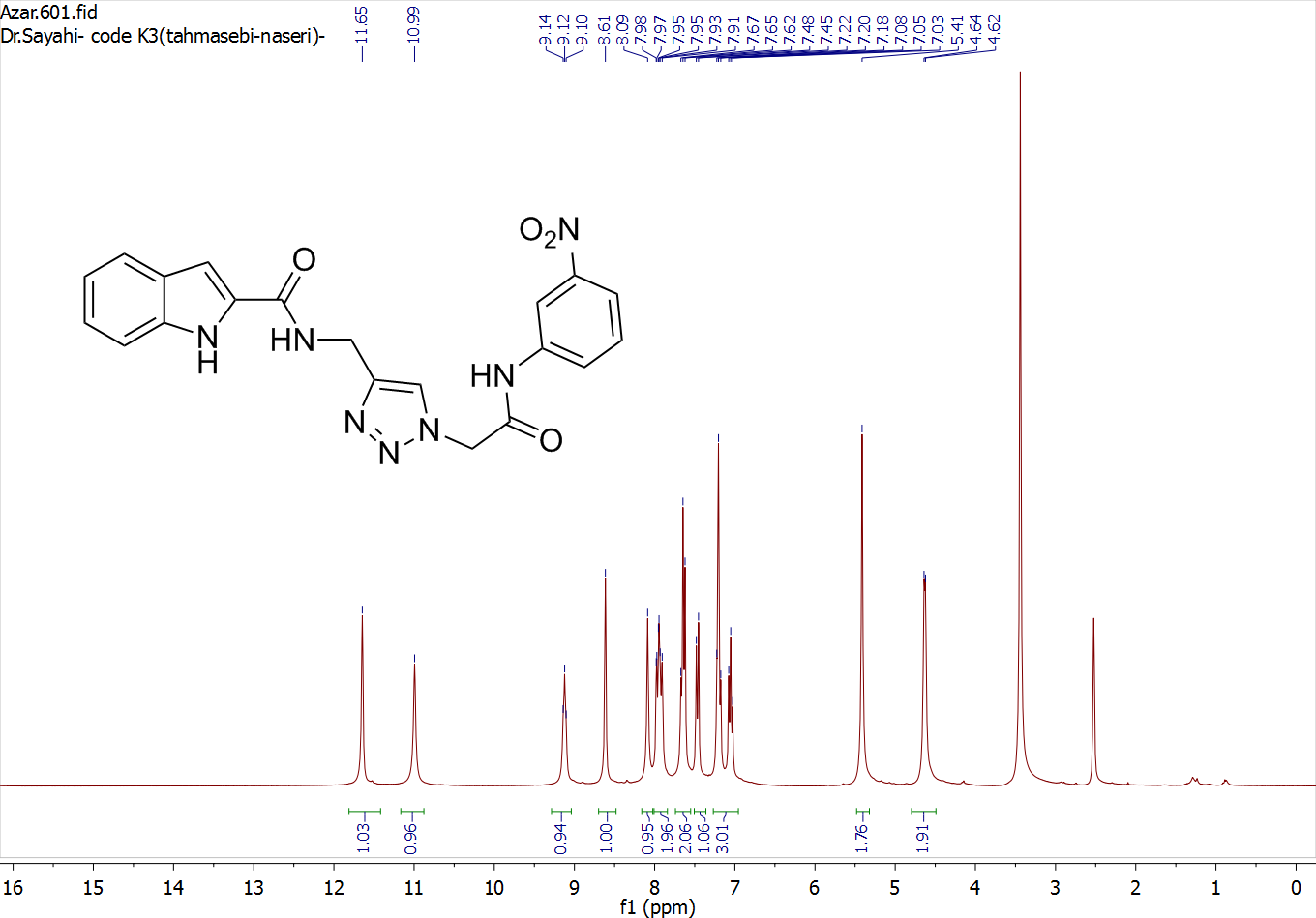
*

*
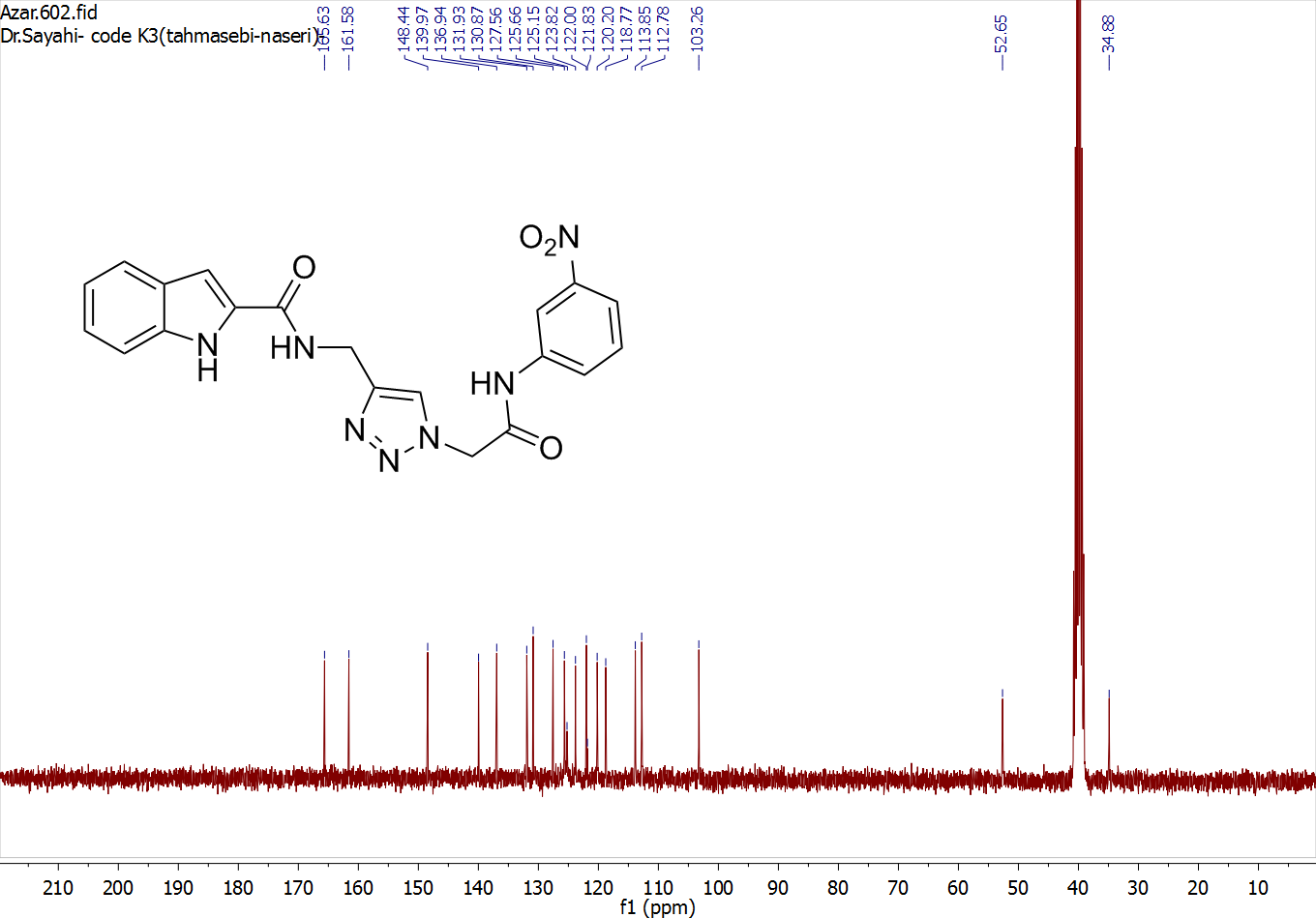
*

*N*-((1-(2-((2,4-dichlorophenyl)amino)-2-oxoethyl)-1*H*-1,2,3-triazol-4-yl)methyl)-1*H*-indole-2-carboxamide (**5j**)

*
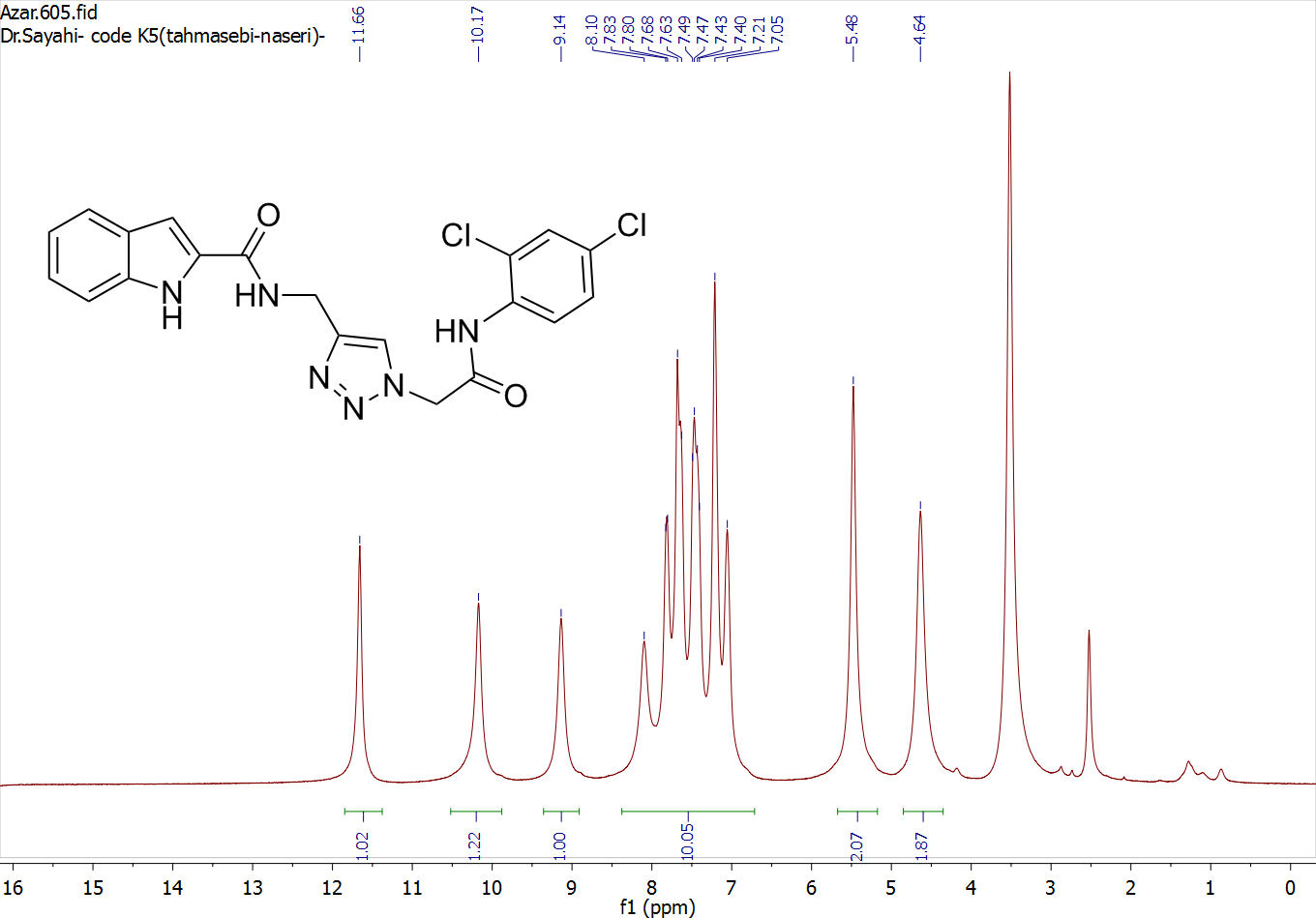
*

*
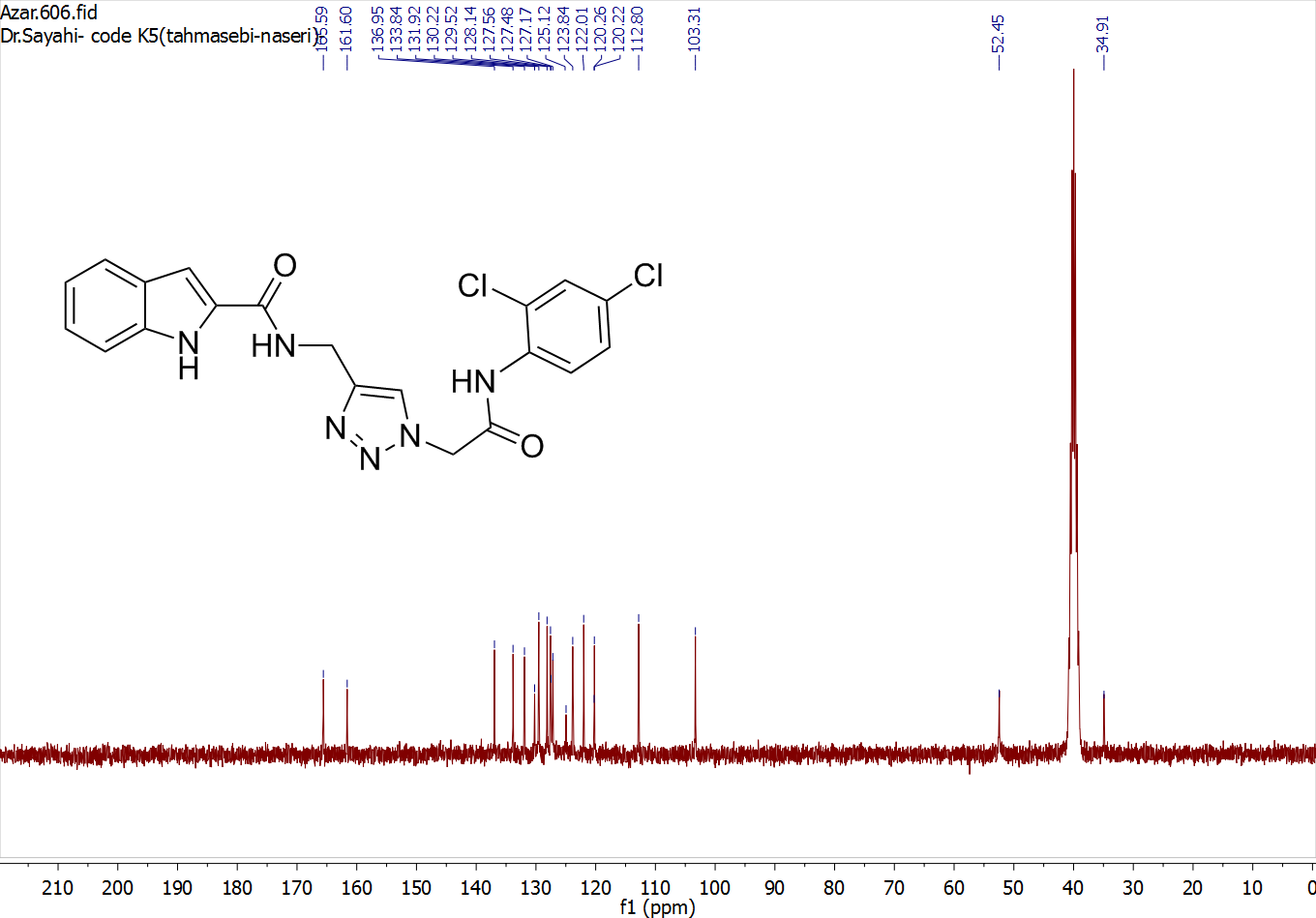
*

*N*-((1-(2-((4-methoxyphenyl)amino)-2-oxoethyl)-1*H*-1,2,3-triazol-4-yl)methyl)-1*H*-indole-2-carboxamide (**5g**)

*
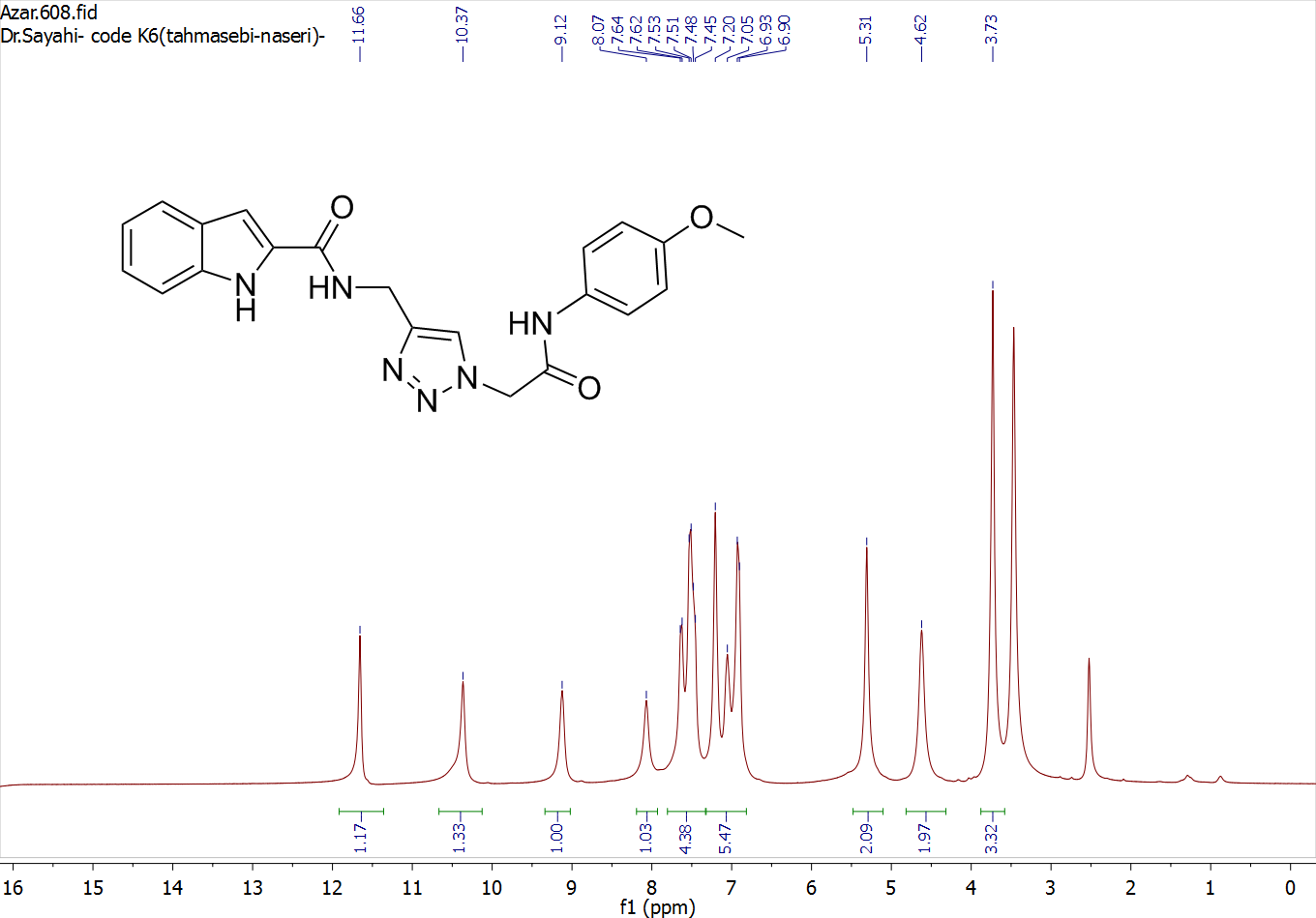
*

*
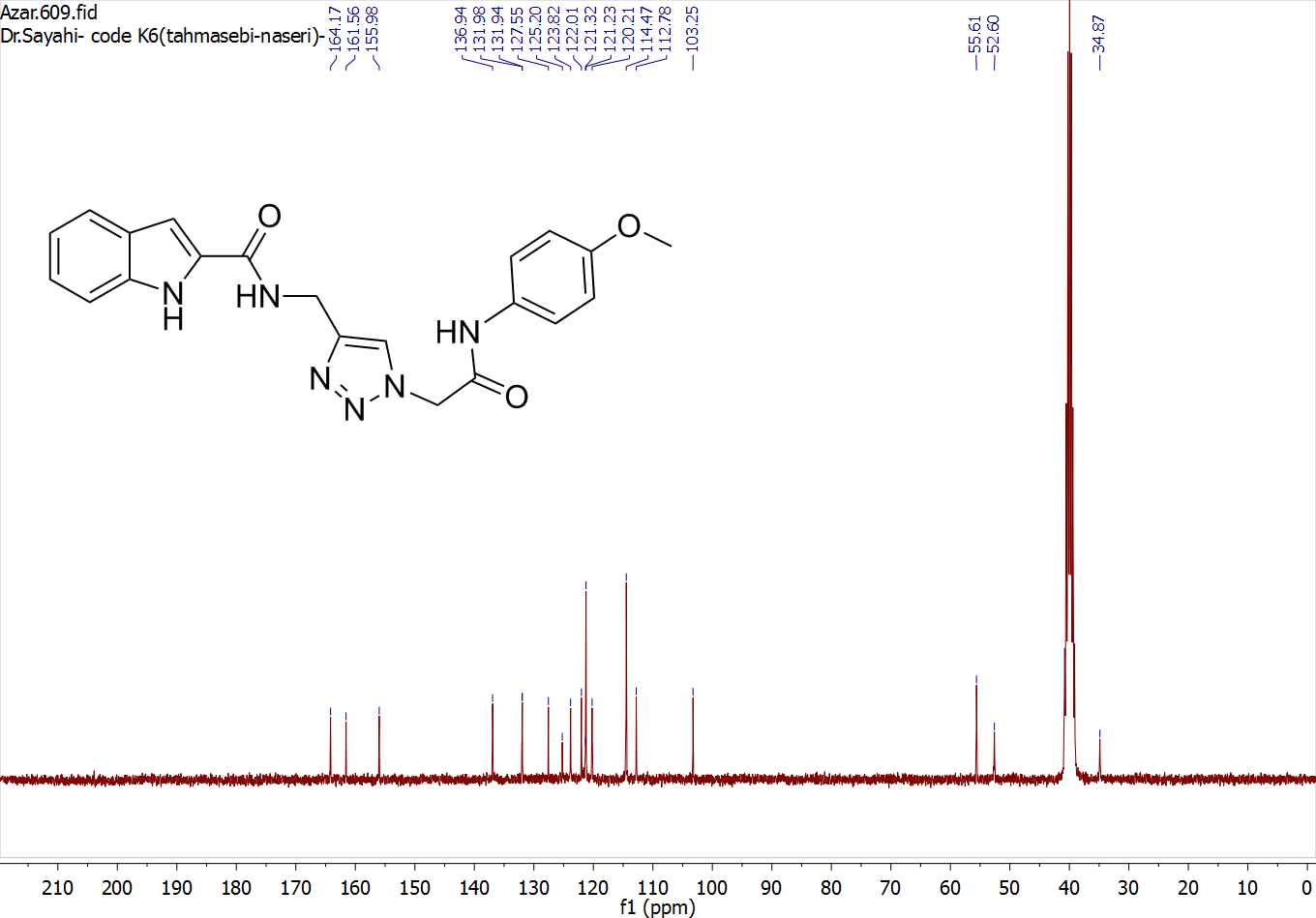
*

*N*-((1-(2-oxo-2-(p-tolylamino)ethyl)-1*H*-1,2,3-triazol-4-yl)methyl)-1*H*-indole-2-carboxamide (**5c**)

*
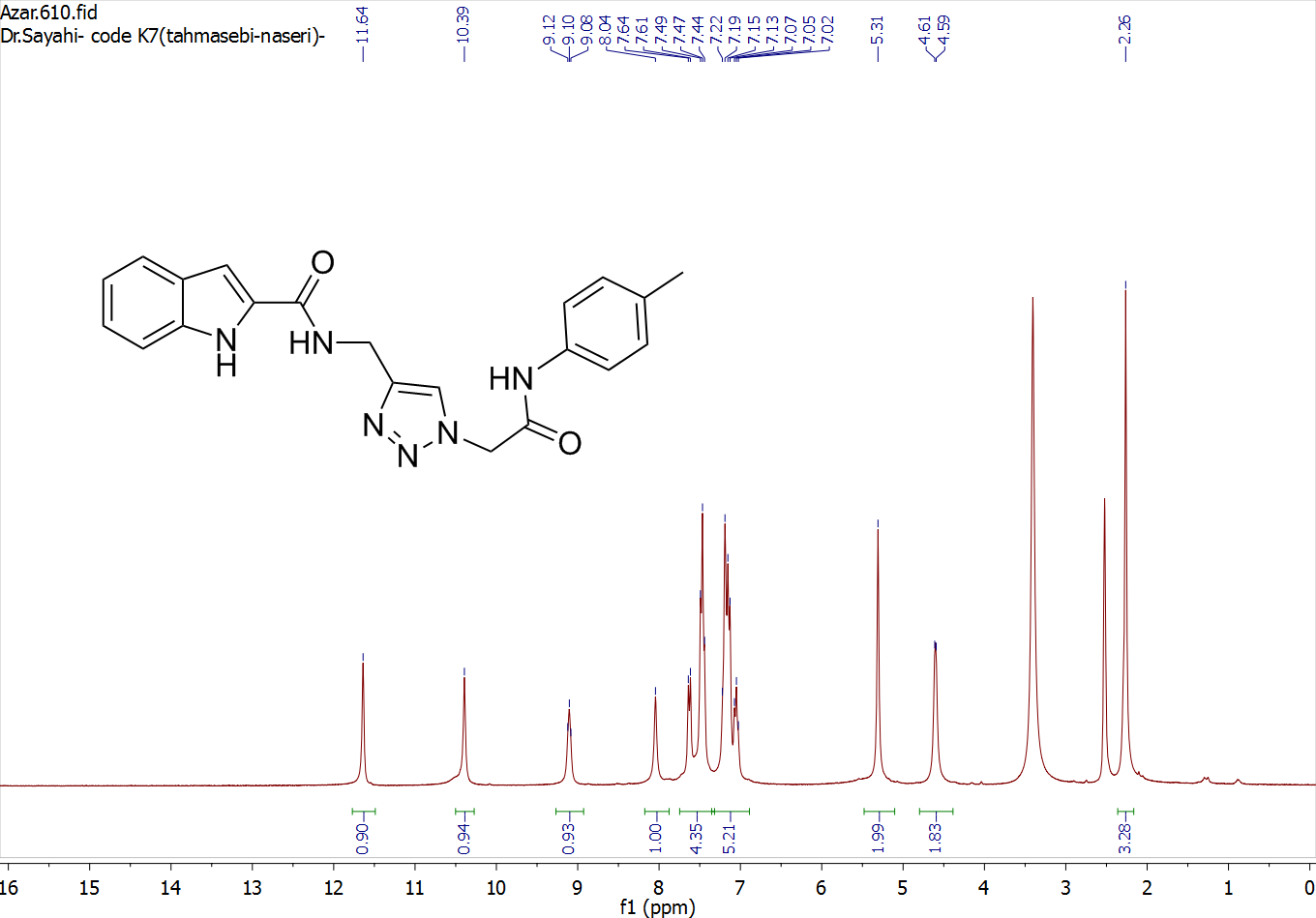
*

*
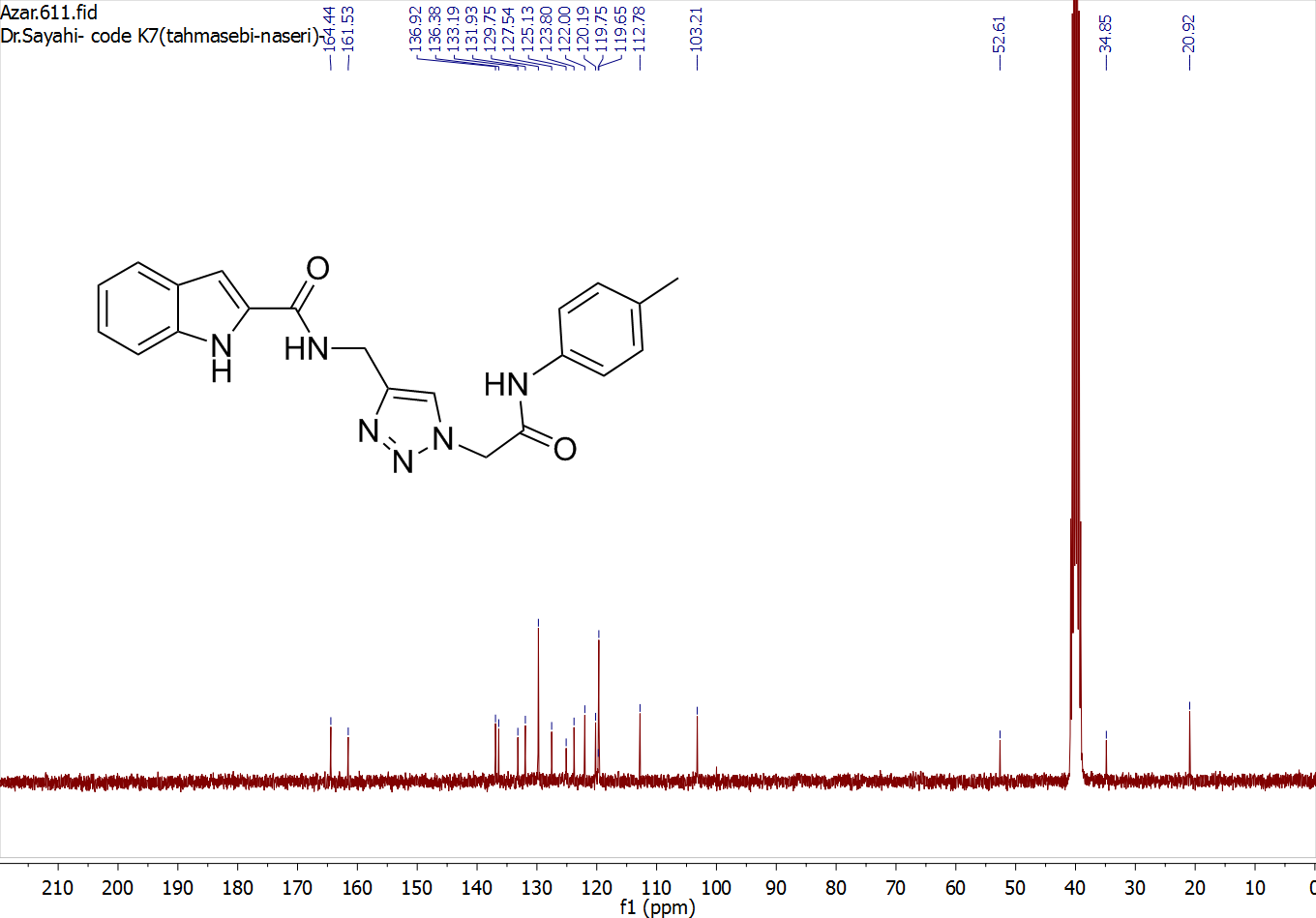
*

*N*-((1-(2-((4-chlorophenyl)amino)-2-oxoethyl)-1*H*-1,2,3-triazol-4-yl)methyl)-1*H*-indole-2-carboxamide (**5i**)

*
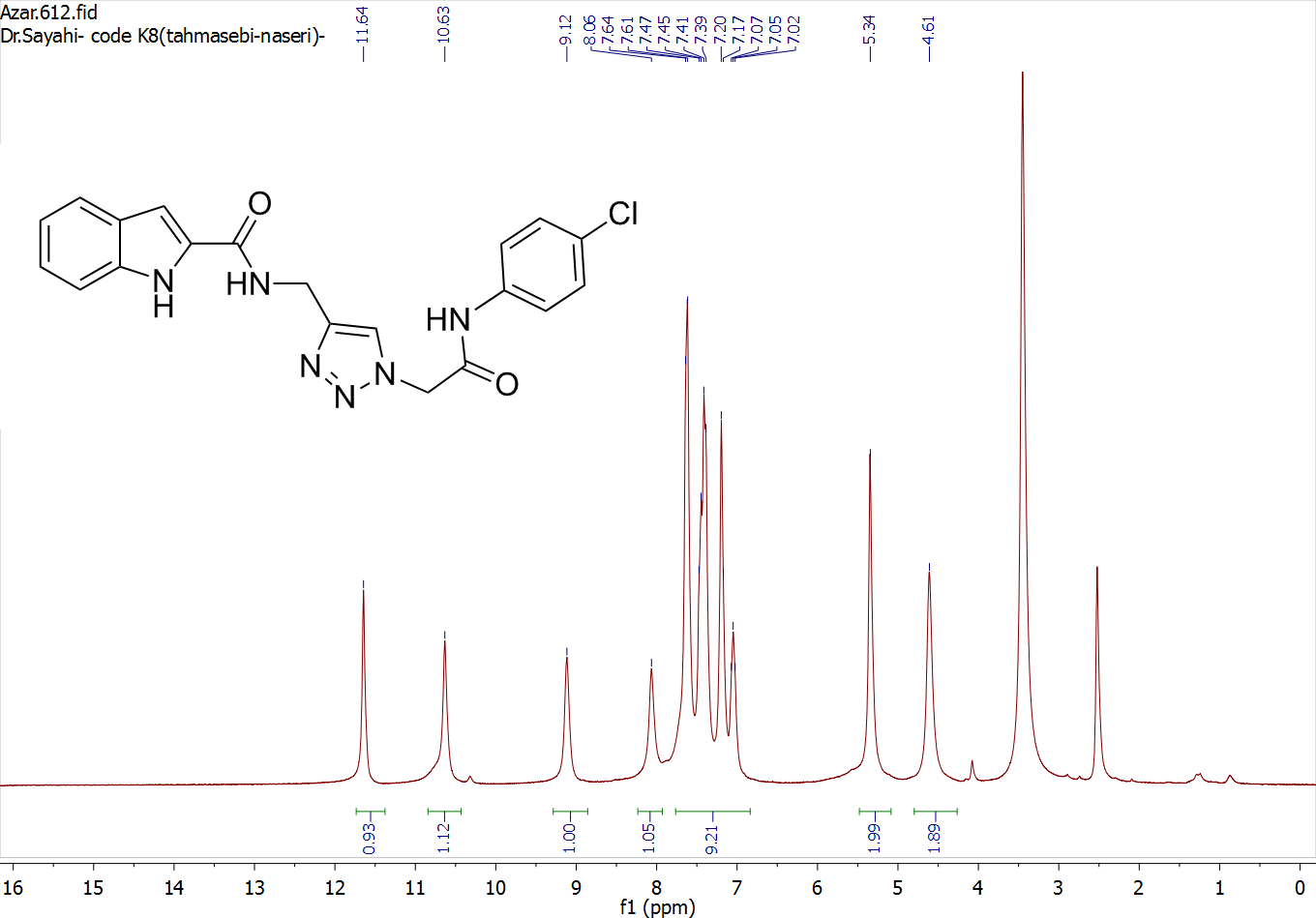
*

*
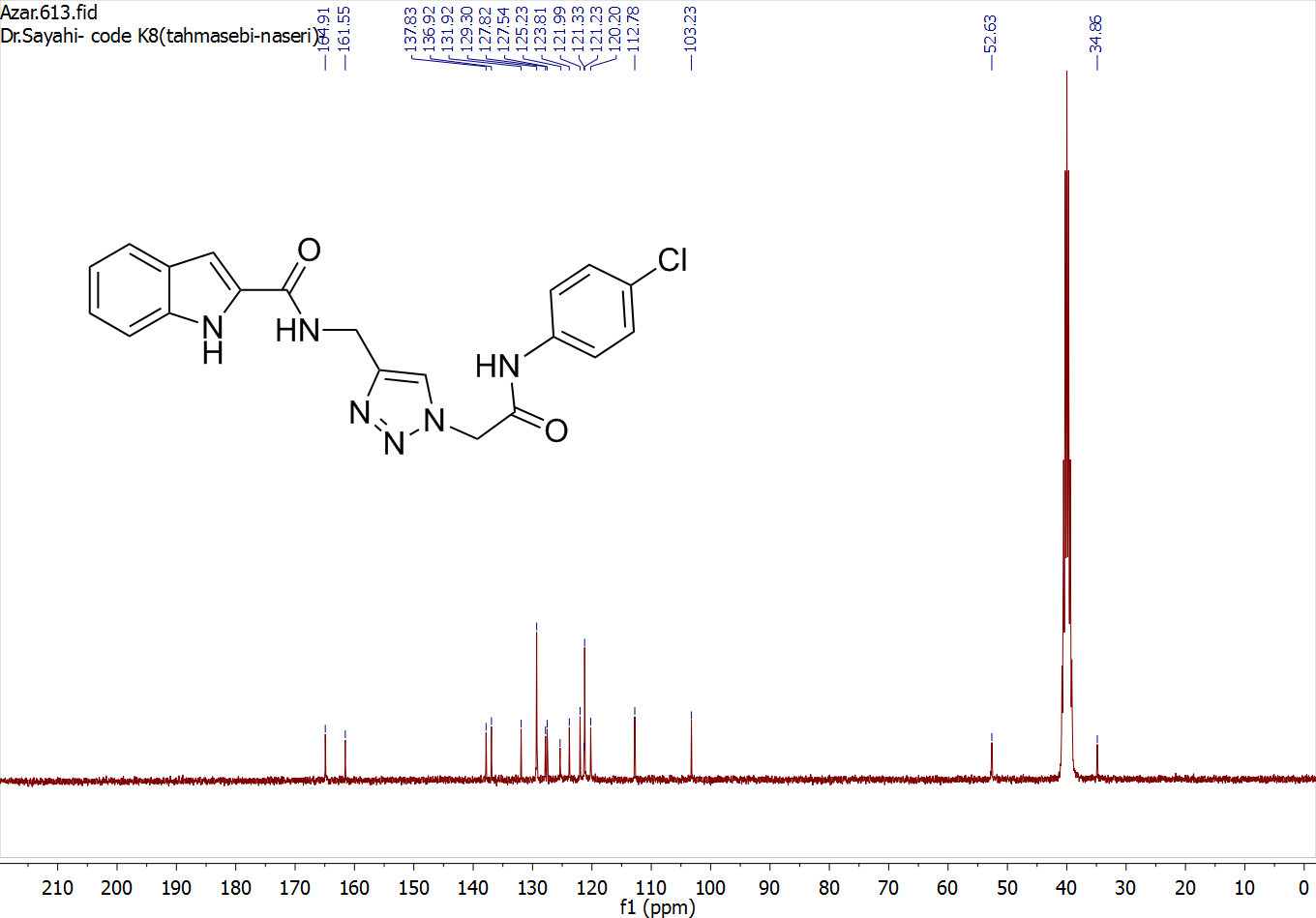
*

*N*-((1-(2-((4-ethylphenyl)amino)-2-oxoethyl)-1*H*-1,2,3-triazol-4-yl)methyl)-1*H*-indole-2-carboxamide (**5f**)


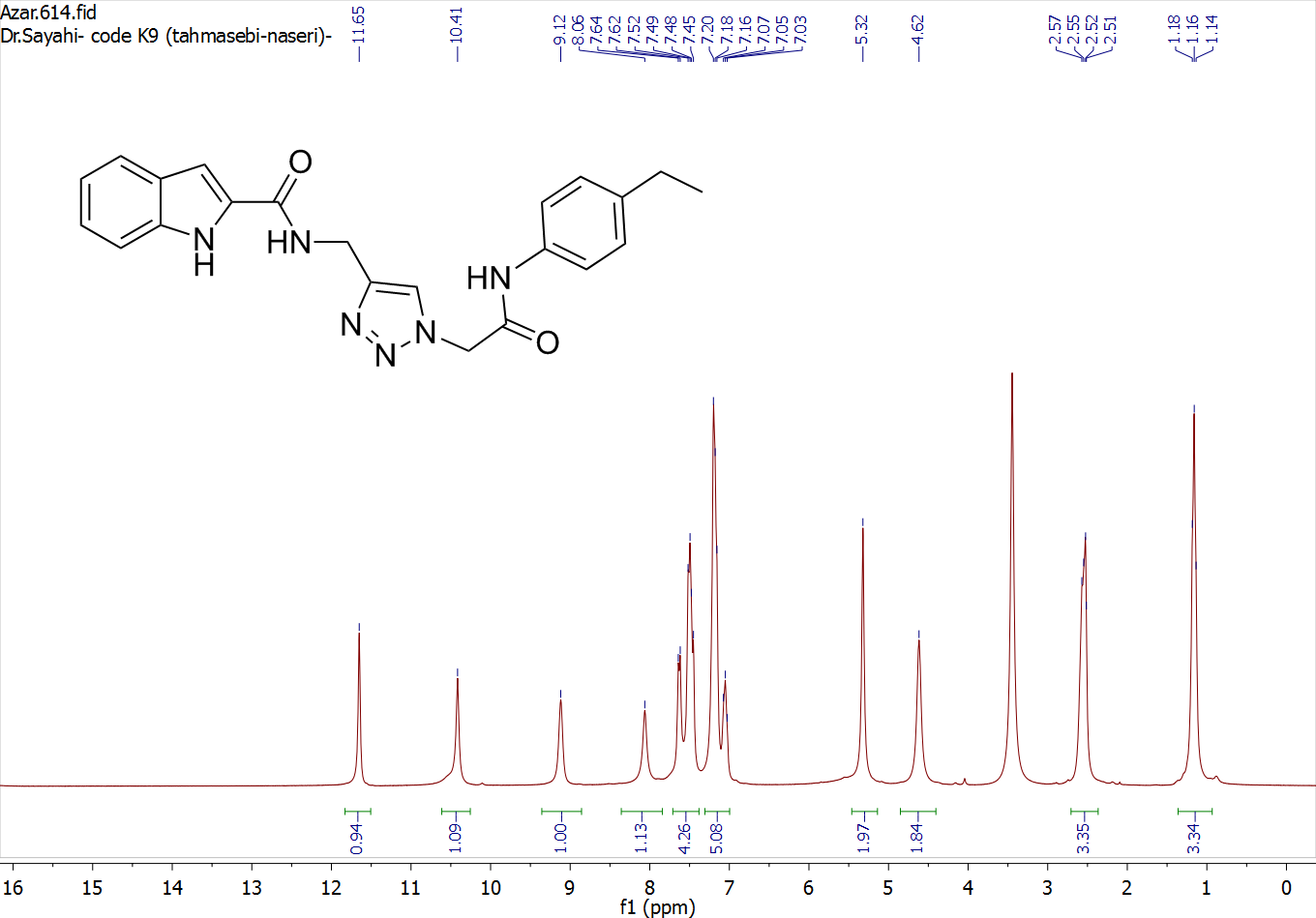


*
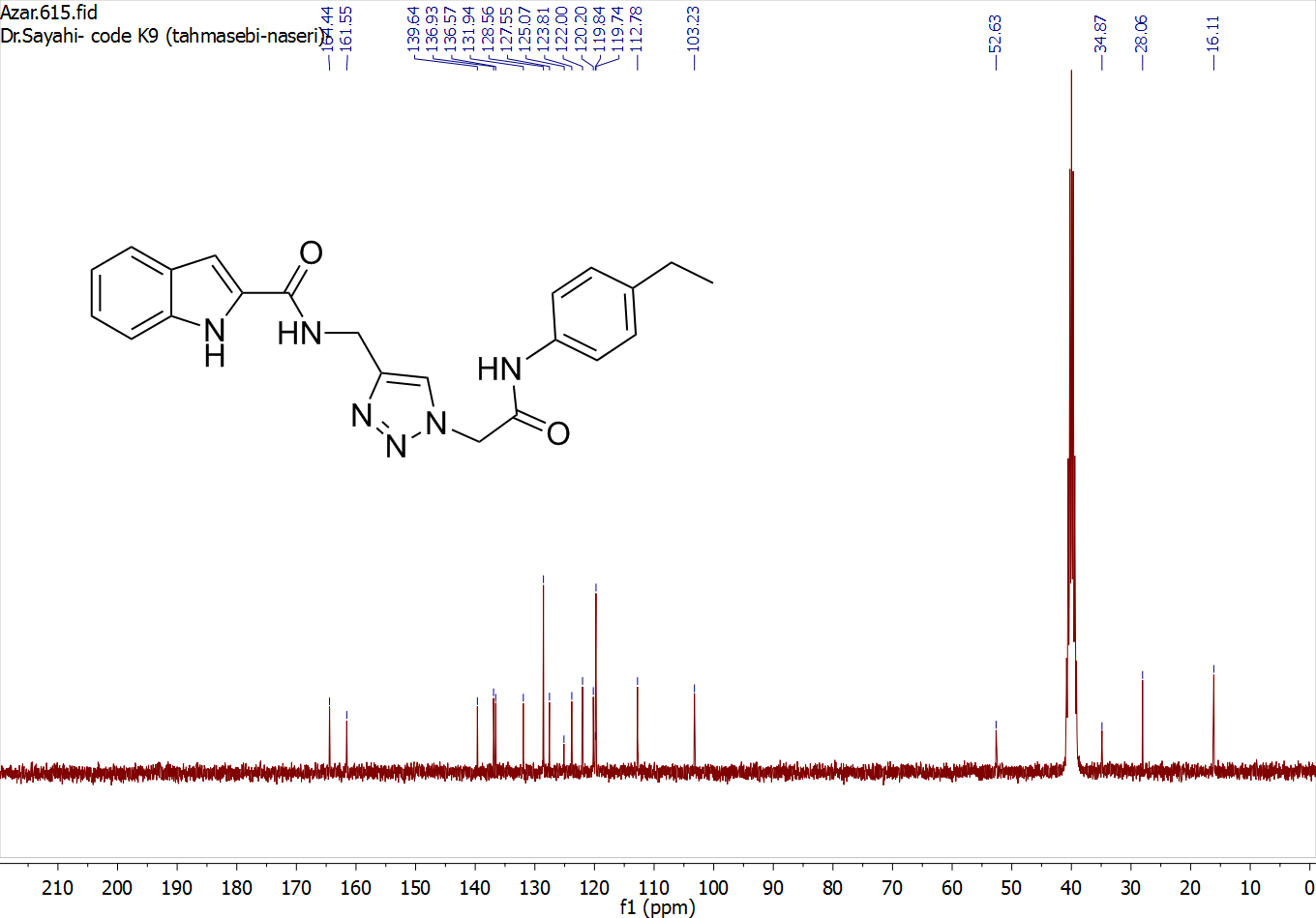
*

*N*-((1-(2-((4-nitrophenyl)amino)-2-oxoethyl)-1*H*-1,2,3-triazol-4-yl)methyl)-1*H*-indole-2-carboxamide (**5m**)


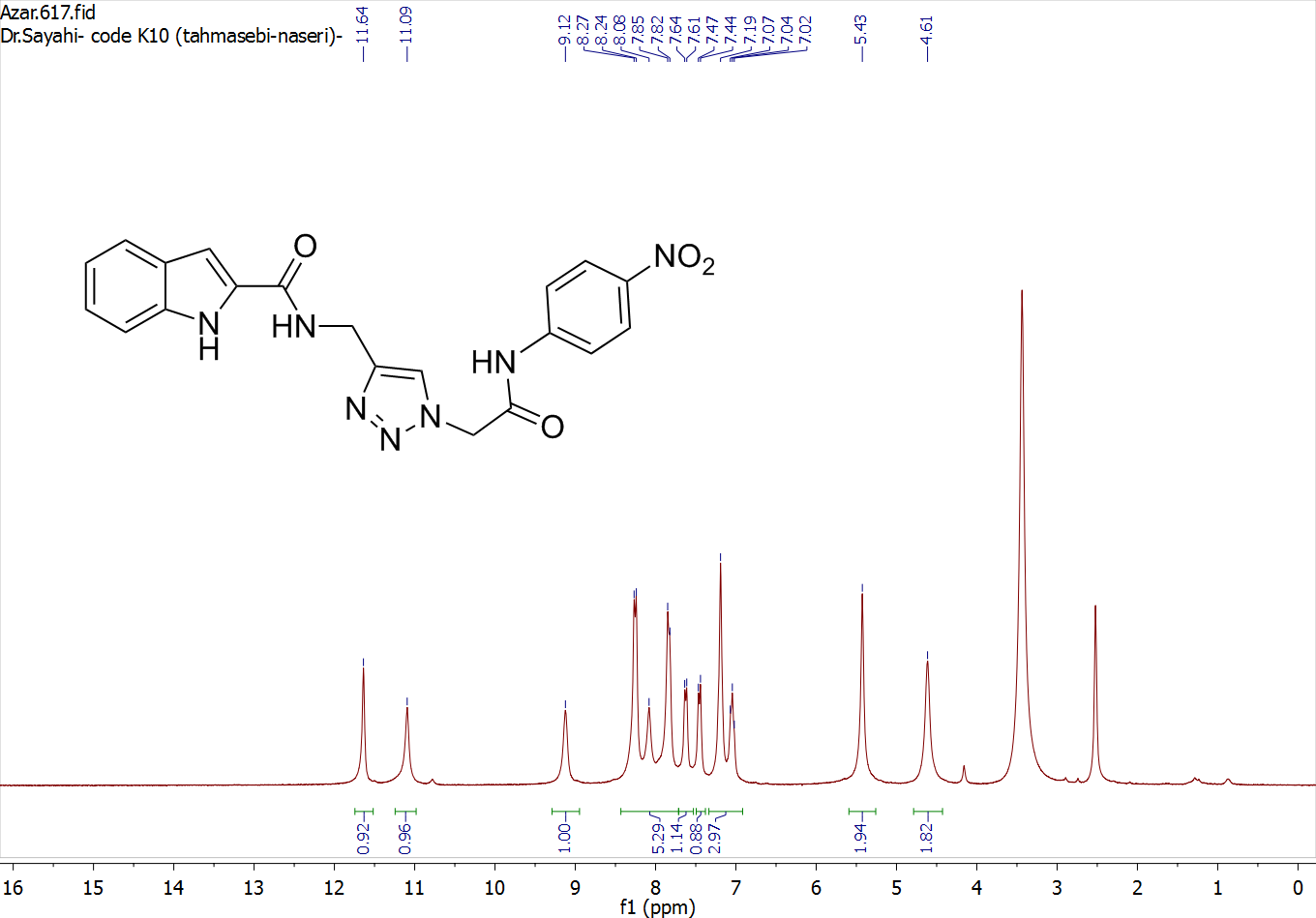


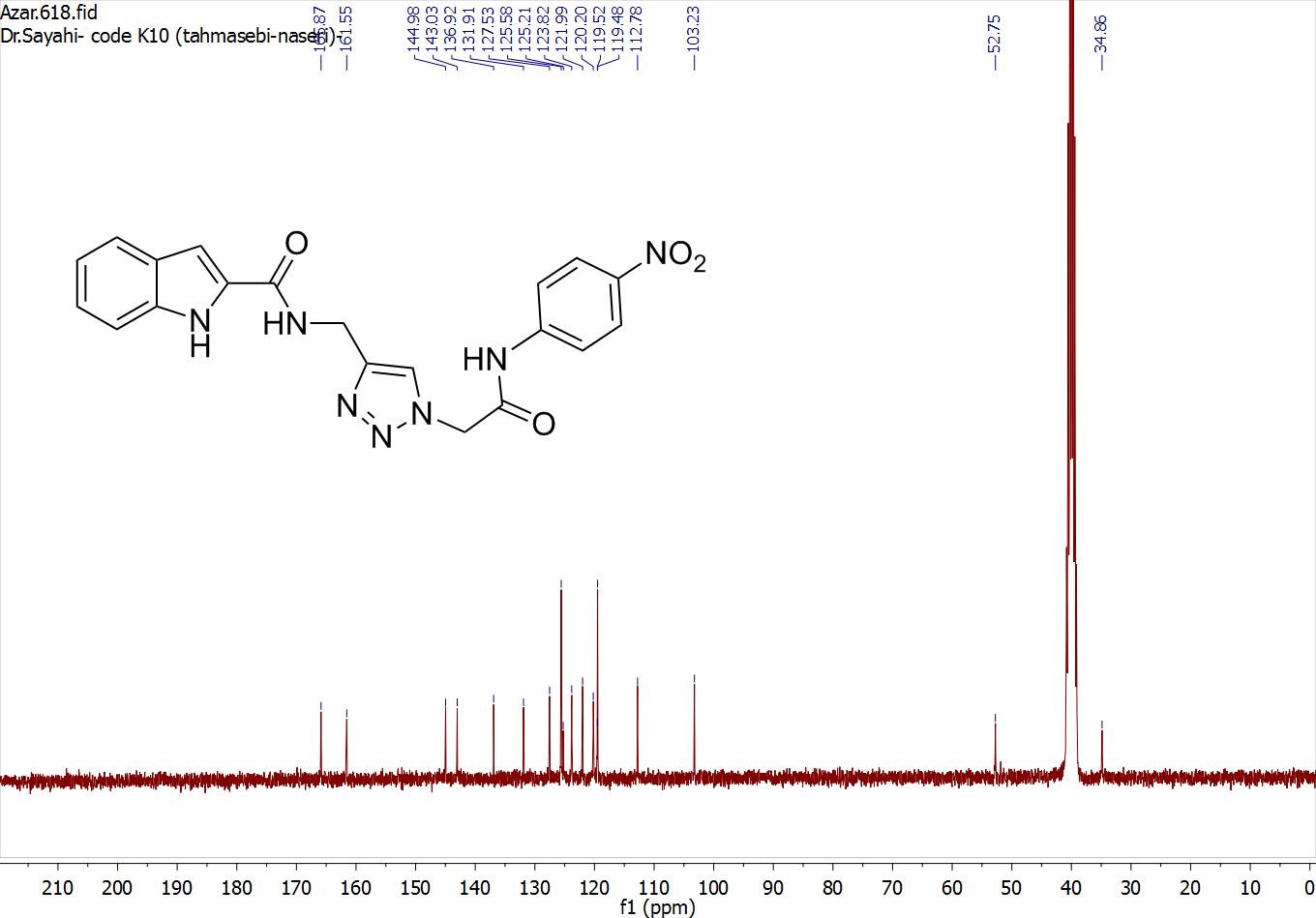


*N*-((1-(2-((2,3-dimethylphenyl)amino)-2-oxoethyl)-1*H*-1,2,3-triazol-4-yl)methyl)-1H-indole-2-carboxamide (**5d**)


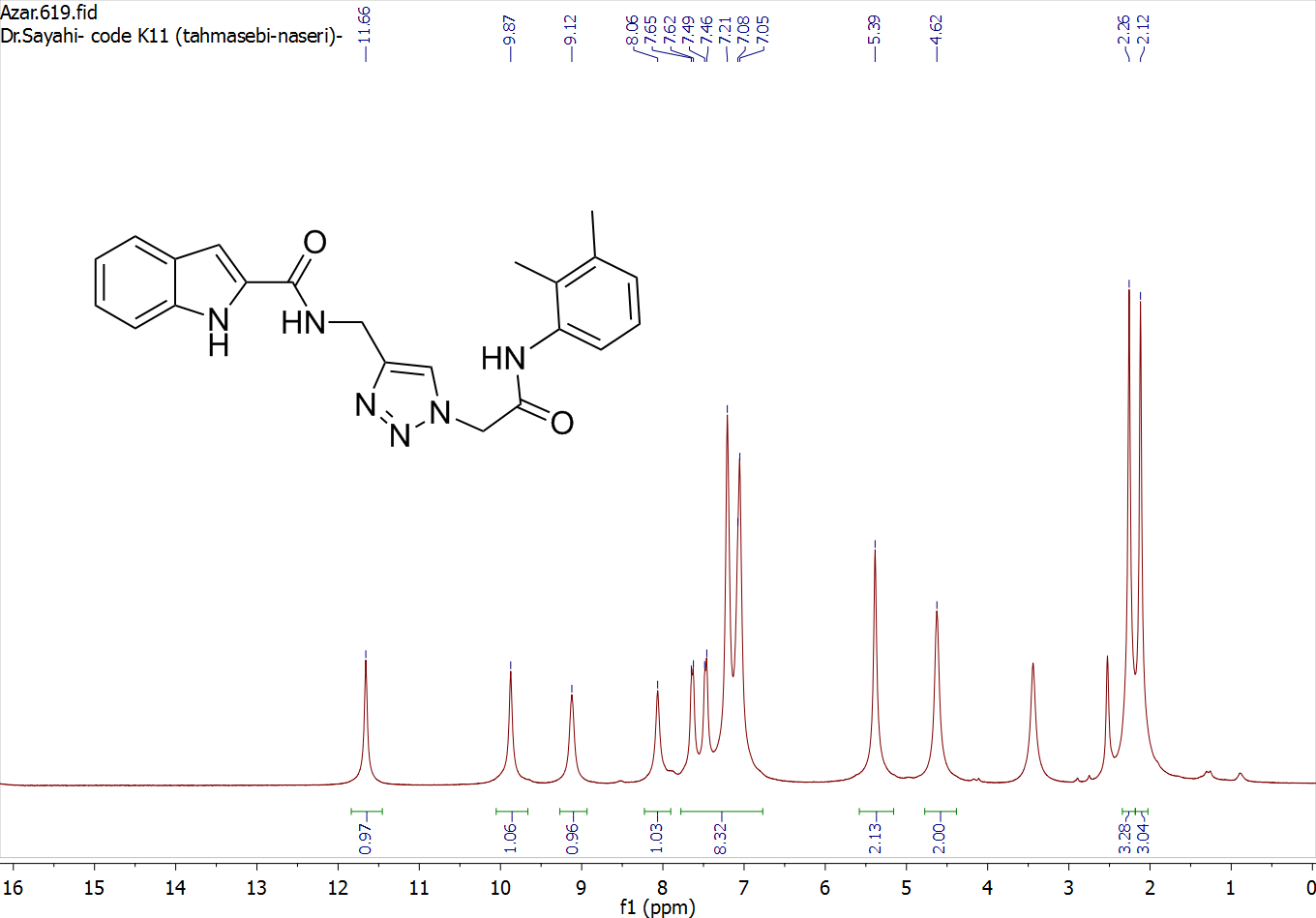


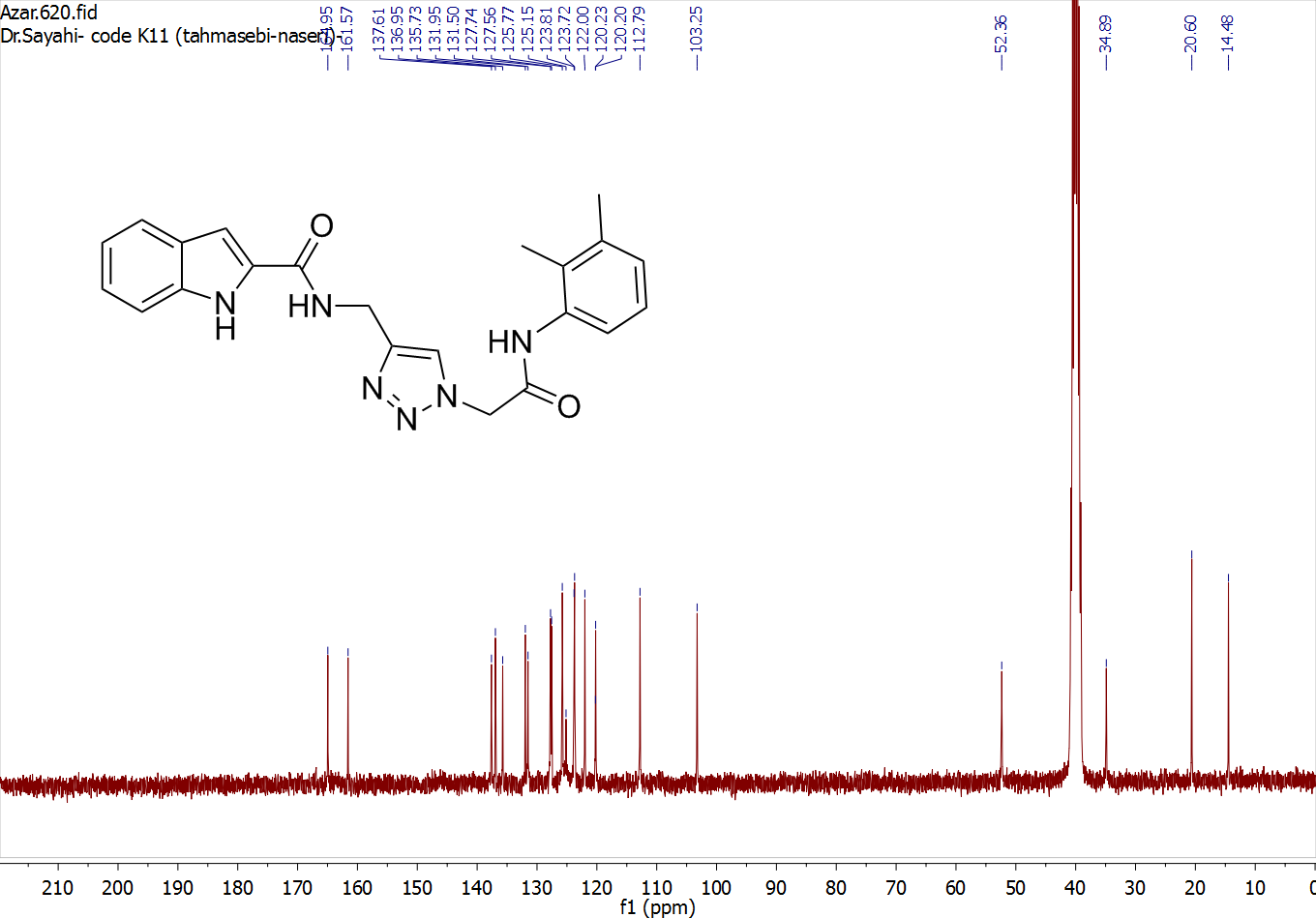


*N*-((1-(2-((2,6-dimethylphenyl)amino)-2-oxoethyl)-1*H*-1,2,3-triazol-4-yl)methyl)-1*H*-indole-2-carboxamide (**5e**)


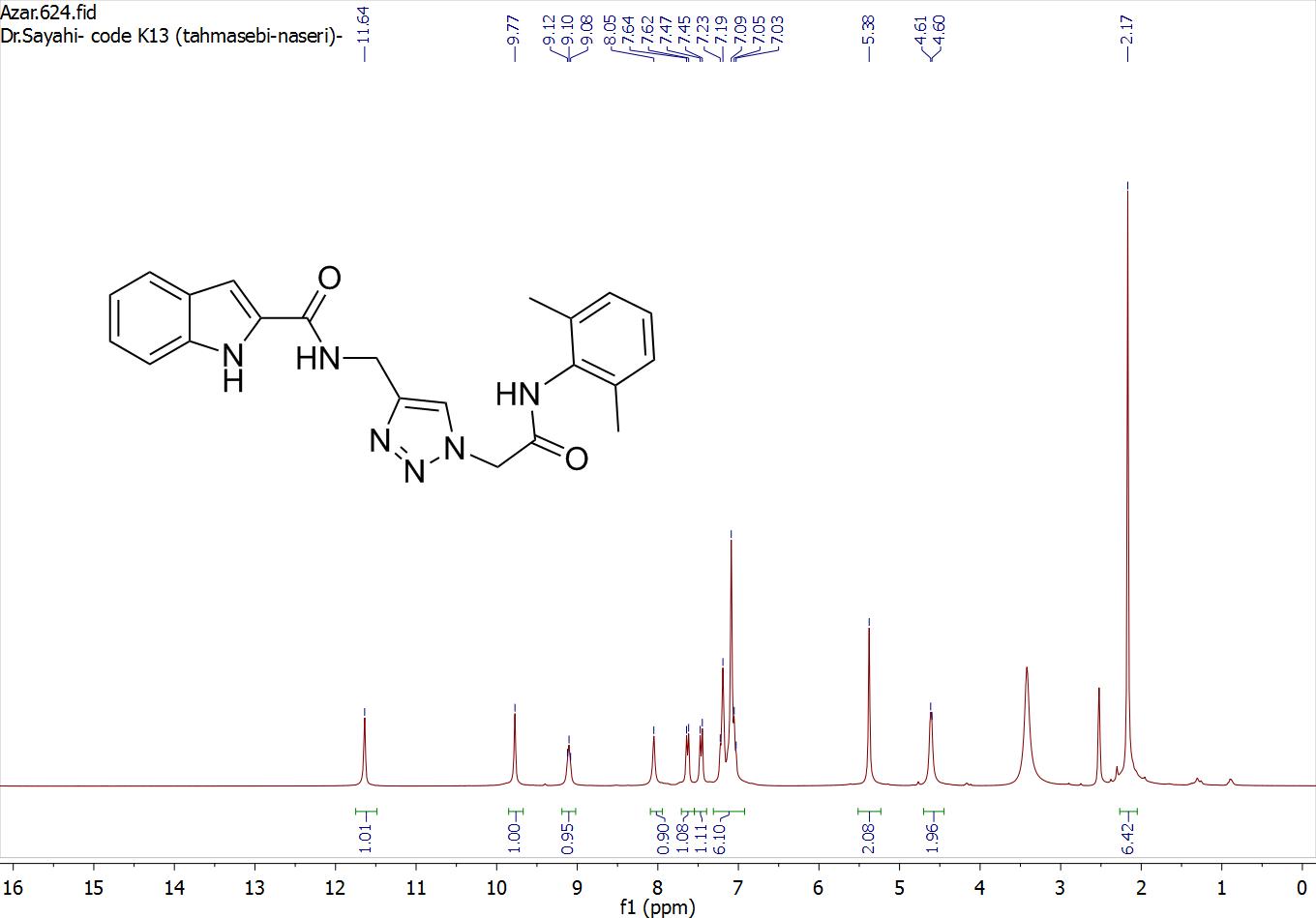


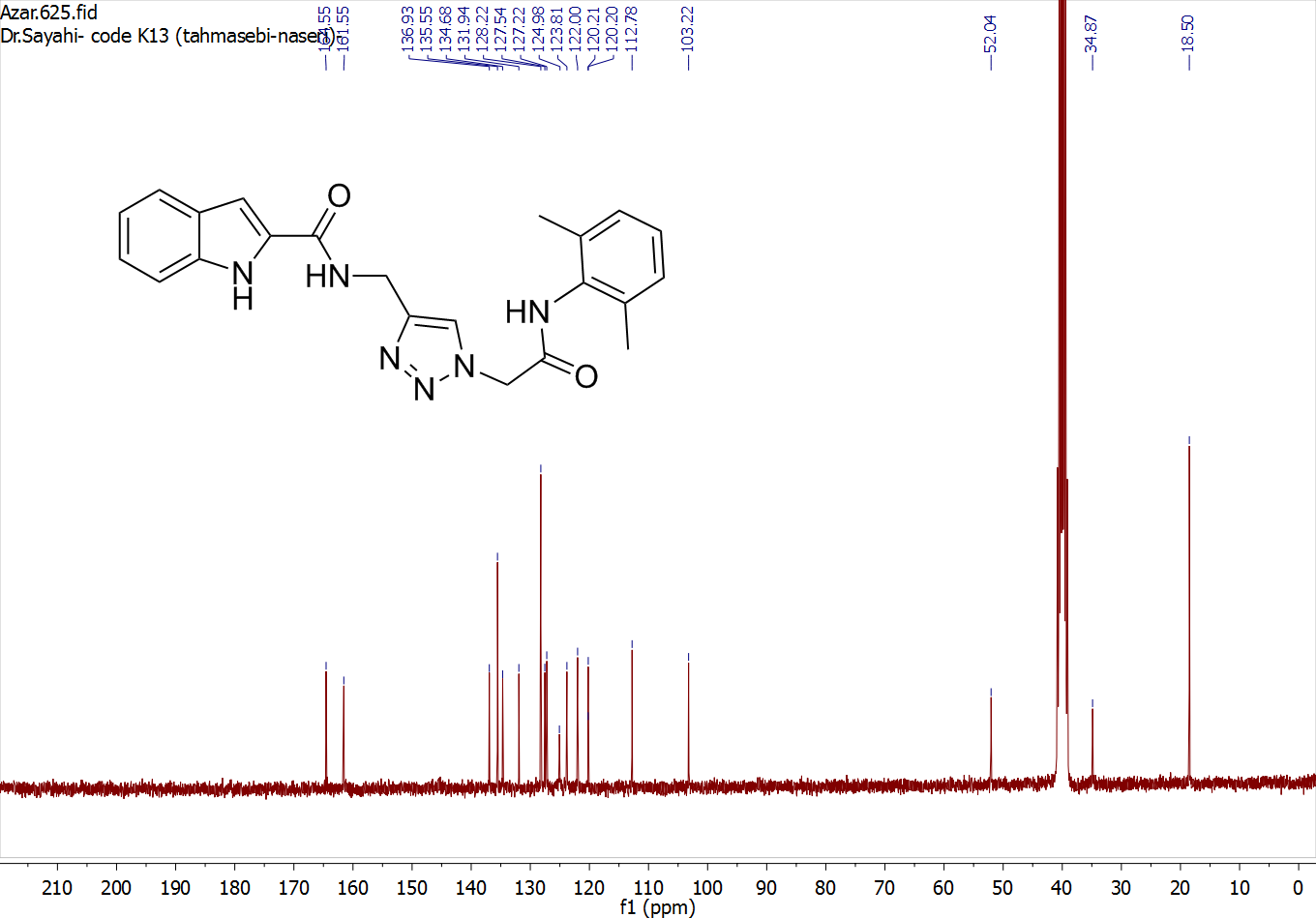


*N*-((1-(2-((2-methyl-4-nitrophenyl)amino)-2-oxoethyl)-1*H*-1,2,3-triazol-4-yl)methyl)-1*H*-indole-2-carboxamide (**5n**)


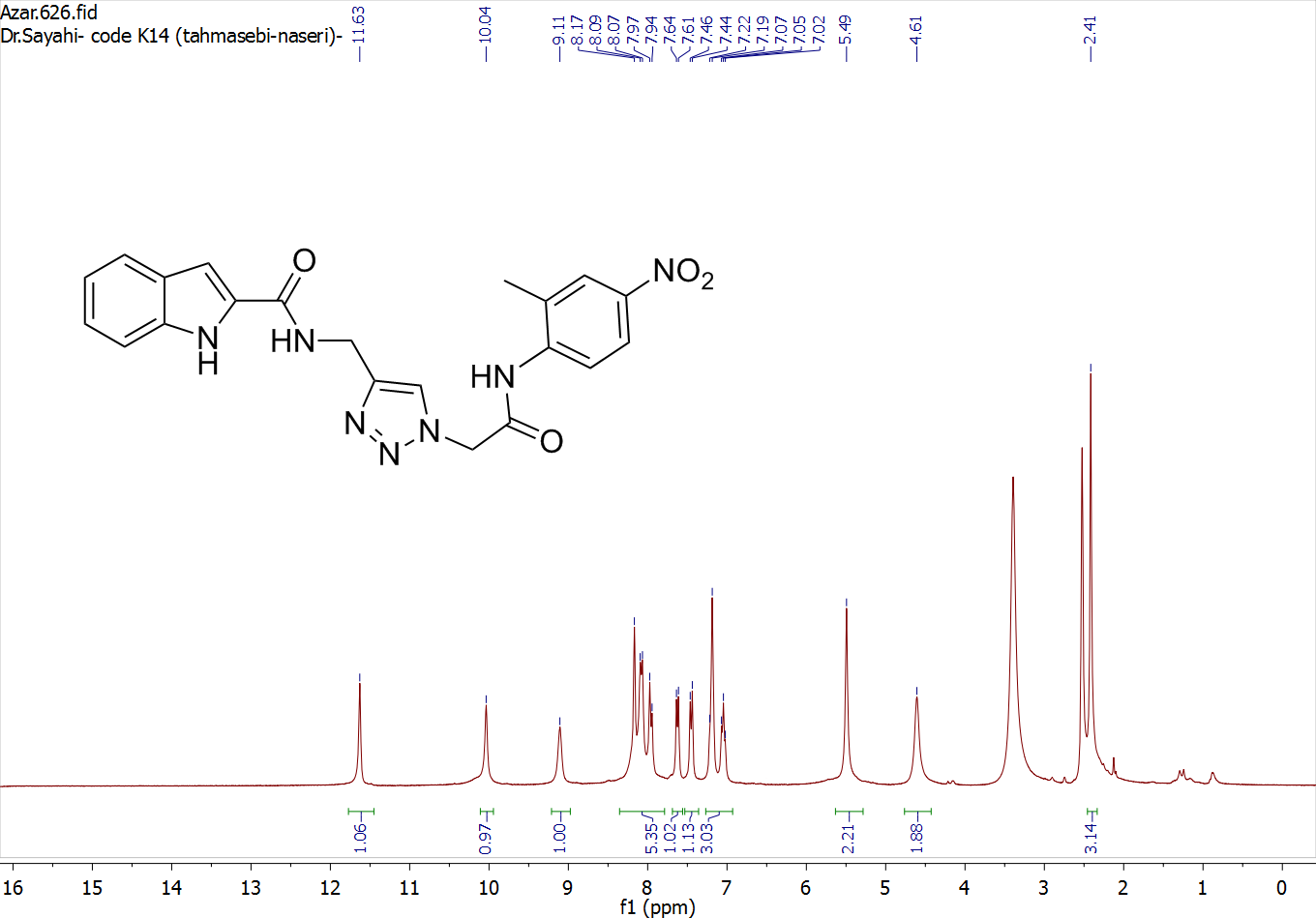


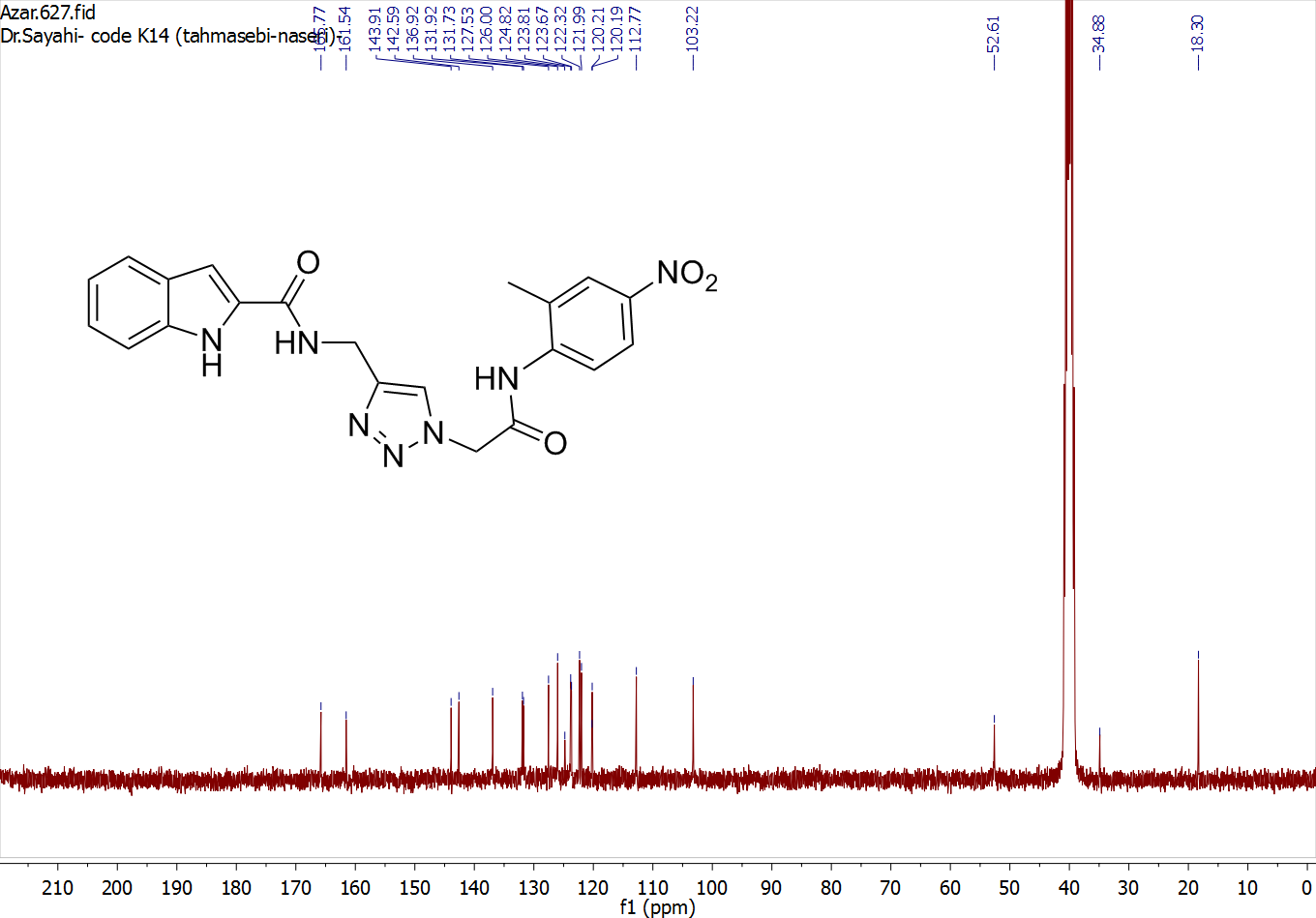


*N*-((1-(2-((3-chlorophenyl)amino)-2-oxoethyl)-1*H*-1,2,3-triazol-4-yl)methyl)-1*H*-indole-2-carboxamide (**5h**)


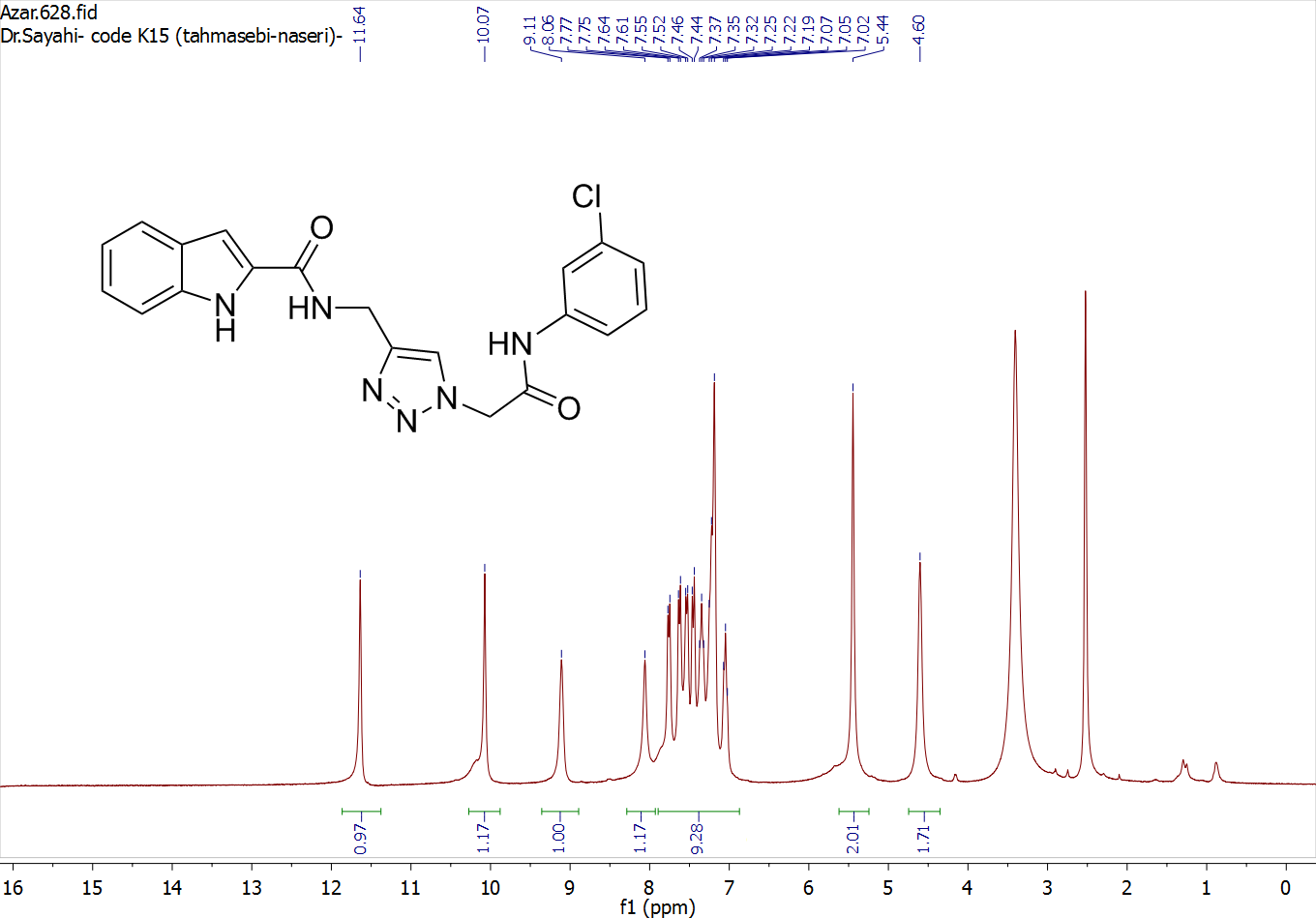


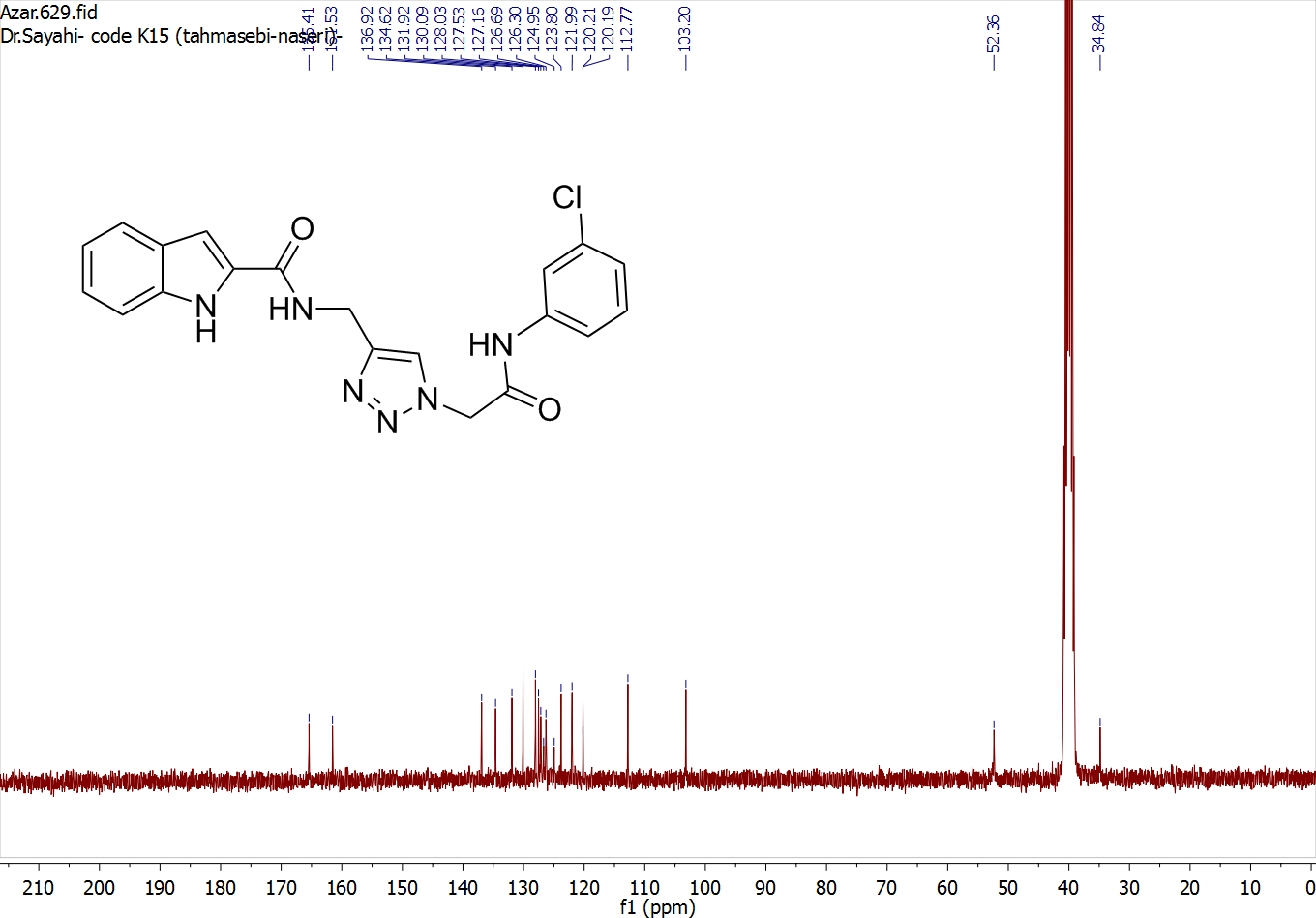

Supplement: Supplementary file 1 — Supplementary Information. [file 41598_2024_66201_MOESM1_ESM.docx]
